# Design, Synthesis and Antibacterial Evaluation of Some New 2-Phenyl-quinoline-4-carboxylic Acid Derivatives

**DOI:** 10.3390/molecules21030340

**Published:** 2016-03-10

**Authors:** Xiaoqin Wang, Xiaoyang Xie, Yuanhong Cai, Xiaolan Yang, Jiayu Li, Yinghan Li, Wenna Chen, Minghua He

**Affiliations:** 1 School of Pharmacy, Guangdong Medical University, Dongguan 523808, China; 13650470904@163.com (X.X.); yuanhong_cai@yahoo.com (Y.C.); yuaishideilu@yahoo.com (J.L.); 13650317182@163.com (Y.L.); cwn201508@163.com (W.C.); 2 Department of Hematology, Donghua Affiliated Hospital of Sun Yat-sen University, Dongguan 523110, China; candyx123@163.com

**Keywords:** 2-phenyl-quinoline-4-carboxylic acid derivatives, synthesis, Doebner reaction, antibacterial activity

## Abstract

A series of new 2-phenyl-quinoline-4-carboxylic acid derivatives was synthesized starting from aniline, 2-nitrobenzaldehyde, pyruvic acid followed by Doebner reaction, amidation, reduction, acylation and amination. All of the newly-synthesized compounds were characterized by ^1^H-NMR, ^13^C-NMR and HRMS. The antibacterial activities of these compounds against Gram-negative (*Escherichia coli*, *Pseudomonas aeruginosa)* and Gram-positive bacteria (*Staphylococcus aureus*, *Bacillus subtilis*), as well as one strain of methicillin-resistant *Staphylococcus aureus* (MRSA) bacteria were evaluated by the agar diffusion method (zone of inhibition) and a broth dilution method (minimum inhibitory concentration (MIC)), and their structure-activity relationships were obtained and discussed. The results revealed that some compounds displayed good antibacterial activity against *Staphylococcus aureus*, and Compounds **5a_4_** and **5a_7_** showed the best inhibition with an MIC value of 64 μg/mL against *Staphylococcus aureus* and with an MIC value of 128 μg/mL against *Escherichia coli,* respectively. The results of the MTT assay illustrated the low cytotoxicity of Compound **5a_4_**.

## 1. Introduction

Bacterial infection is one of the most complex global health issues of this century [1]. Furthermore, many drug-resistant pathogens have emerged in recent years because of the increasing use or abuse of antibacterial agents [2,3], and antibiotic drug resistance has become a growing problem across the globe in recent years [4]. Currently, many advances for combating antimicrobial resistance have been achieved, such as modifications/improvements of existing antibiotic classes, some anti-adhesion agents against Gram-positive pathogens [5] and efflux pump inhibitors (EPIs) [6]. However, this problem has not been completely resolved. Therefore, the development of novel and effective chemotherapeutics is still in demand to overcome these problems [7,8].

**Figure 1:**
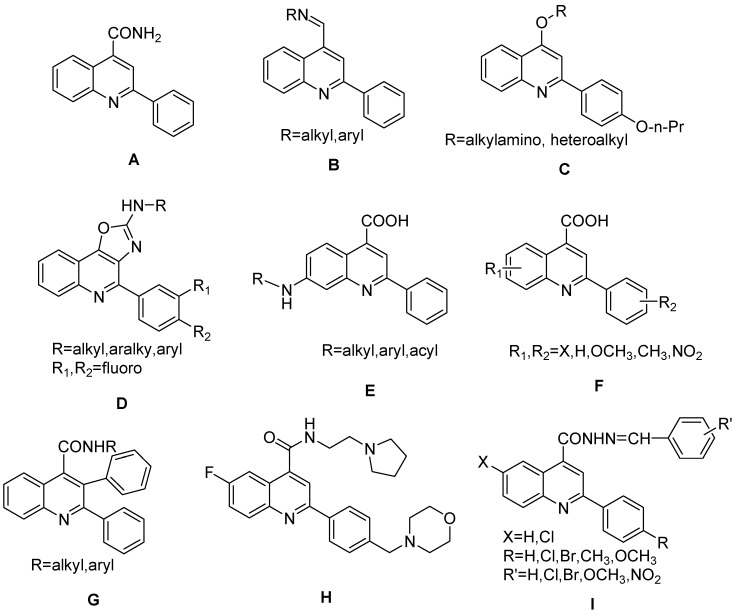
The structures of 2-aryl-quinoline-4-carboxylic acid derivatives with antimicrobial activities.

The quinoline ring occurs in various natural products and represents a key motif in medicinal chemistry [9,10,11,12], and its derivatives, quinoline-4-carboxylic acids, are a group of compounds associated with different biological activities, such asantiviral [13], anti-inflammatory [14], antimicrobial [15], anti-atherothrombosis [16], antiemetic [17], anxiolytic [18], antimalarial and antileishmanial [19]. Although quinoline-4-carboxylic acid derivatives exhibit various bioactivities, the research of new quinoline-4-carboxylic acid-based antibacterial drugs has developed slowly. Some quinoline-4-carboxylic acid derivatives exhibited certain antibacterial activity [20,21]. Furthermore, a literature review revealed that the presence of an aryl ring at the second position of quinoline-4-carboxylic acid derivatives exhibits good antibacterial activity for the target compound and plays a significant role in the development of new antibacterials (Figure 1) [6,15,22,23,24,25,26,27], and they are very suitable for further modifications to obtain more effective antibacterial agents. In addition, the structure-activity relationship (SAR) studies of quinolones have indicated that the introduction of a basic group in the quinoline is useful for regulating the physicochemical property and influencing their potency, spectrum and safety [28,29]. All of these stimulated us with great interest to focus on further structural modification of quinoline-4-carboxylic acid in order to find novel derivatives with potential activity against bacterial strains.

Keeping in view the wide range of pharmaceutical activities of quinoline-4-carboxylic acid, in this report, we introduced two amino substituents in the molecule, which may severe as new hydrogen bond donors and receptors to increase binding affinity to the enzyme and also improve their physicochemical properties. Thus, a series of 2-(2’-substutited)-phenyl-quinoline-4-carboxylic acid derivatives was designed, synthesized and evaluated for antibacterial activities.

## 2. Results and Discussion

### 2.1. Chemistry

**Scheme 1:**
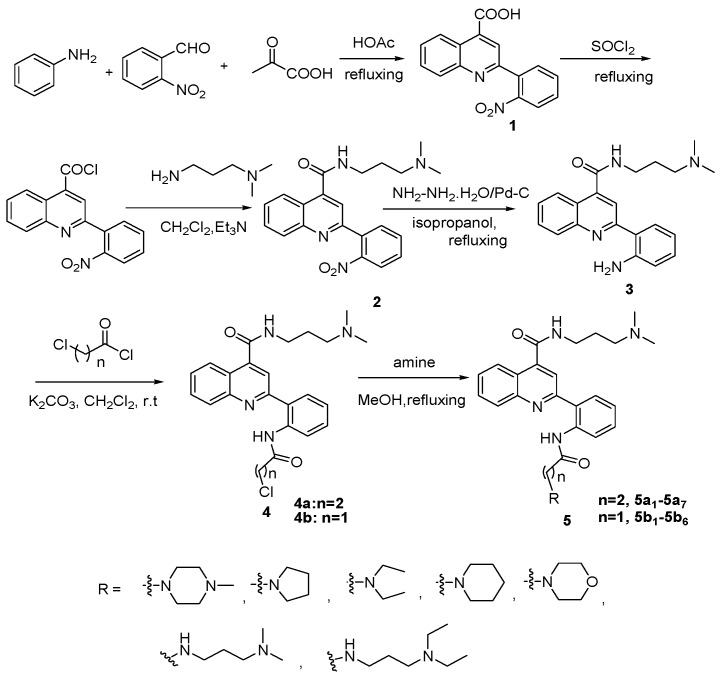
Synthetic route of 2-phenyl-quinoline-4-carboxylic acid derivatives.

The synthetic pathway for the target Compounds **5a_1_**–**5a_7_** and **5b_1_**–**5b_6_** is depicted in Scheme 1. The intermediate 2-(2-nitrophenyl)-quinoline-4-carboxylic acid (**1**) is considered to be a category of versatile intermediates that provide building blocks in the synthesis of poly-substituted quinoline-4-carboxylic acid derivatives. Several methods have been developed to date for the synthesis of 2-(2-nitrophenyl)-quinoline-4-carboxylic acid, and these include the Doebner reaction using aniline, 2-nitrobenzaldehyde, pyruvic acid and a catalytic amount of trifluoroacetic acid in ethanol media [14,15], as well as the versatile Pfitzinger reaction by reacting isatin with α-methyl ketone in aqueous ethanol [22,30,31]. 2-(2-nitrophenyl)-quinoline-4-carboxylic acid was also prepared via Doebner reaction under microwave [25,26,32,33]. We opted for the Doebner reaction and explored the influence of two catalysts on the reaction. Treatment of aniline, 2-nitrobenzaldehyde, pyruvic acid and a catalytic amount of trifluoroacetic acid in ethanol media with refluxing for 12 h afforded only a few products, and the pure product must be obtained by flash column chromatography. However, the replacement of trifluoroacetic acid with acetic acid and excessive acetic acid as the solvent instead of ethanol favored the reaction. After completion of the reaction, the mixture was poured into ice water, and the crude product was purified by acid-alkali neutralization and further by recrystallization without flash column chromatography with a total yield of 68%.

The reaction of intermediate **1** with SOCl_2_ under reflux furnished the product acyl chloride. Subsequently, the treatment of intermediate acyl chloride with 3-(dimethylamino)-1-propylamine in the presence of triethylamine gave Compound **2**. In order to obtain the amine functionality, the conventional method for the reduction of the nitro group was attempted by using 80% hydrazine hydrate in the presence of 10% Pd/C and isopropanol as a solvent under reflux, which furnished amino-substituted Compound **3** in a 92% yield [34]. The advantage of this reaction was that product isolation could be facilitated without purification using chromatography. Reaction of amine **3** with acyl chloride and potassium carbonate in dichloromethane gave Compounds **4a** and **4b**. Because of their poor solubilities, the crude product directly was used in the next reaction without purification. Finally, the target Compounds **5a_1_**–**5a_7_** and **5b_1_**–**5b_6_** were obtained through the reaction of Compounds **4a** and **4b** with different primary and secondary amines under reflux conditions for 10 h, respectively.

The structures of the target compounds were validated by using ^1^H-NMR, ^13^C-NMR and HRMS, and their purities were determined to be above 95% by using HPLC.

### 2.2. Evaluation of Antibacterial Activity

Firstly, the newly-synthesized Compounds **5a_1_**–**5a_7_**, **5b_1_**–**5b_6_** were screened for their *in vitro* antibacterial activities by the agar diffusion method against a variety of different strains [35,36,37], including *Staphylococcus aureus* (*S. aureus*), *Bacillus subtilis* (*B. subtilis*), *Escherichia coli* (*E. coli*), *Pseudomonas aeruginosa* (*P. aeruginosa*) and one strain of methicillin-resistant *Staphylococcus aureus* (MRSA) bacteria. Their activities were compared to that of Compound **1** and the known antibacterial agents ampicillin and gentamycin. The results are presented in Table 1.

The antibacterial primary screening results revealed that most of the tested compounds showed moderate inhibition against various tested bacterial strains compared to the standard drug. However, most of them exhibited better activity than Compound **1** against the above-mentioned bacteria; this indicated that the structural modifications of 2-phenyl-quinoline-4-carboxylic acid increased the antibacterial activity. Among the synthesized compounds, Compound **5a_4_** showed significant antibacterial activity against *S. aureus* and *B. subtilis*; Compound **5a_7_** was the most active antibacterial activity against *E. coli*; and Compound **5b_4_** exhibited moderate antibacterial activity against MRSA, but all compounds showed weak inhibition action against *P. aeruginosa.* The LogP values of all of the compounds were predicted on the basis of a computational study using ACDLabs/ChemSketch software. Among the active compounds against all of the mentioned bacteria, it can be clearly seen that the compound (**5a_4_**, LogP = 2.26; **5a_7_**, LogP = 1.94; **5b_4_**, LogP = 2.32) with higher lipophilicity displayed higher activity.

Furthermore, with the same basic terminus, Compounds **5a_1_**–**5a_7_** with longer side chains (*n* = 2, with three bonds between the basic N terminus and carbonyl group) showed stronger antibacterial activity than Compounds **5b_1_**–**5b_6_** with shorter side chains (*n* = 1, with two bonds between the basic N terminus and carbonyl group) against the above-mentioned bacterial strains, respectively. These indicated that the length of the amide side chain at the ortho-position of the 2-phenyl group had a significant effect on antibacterial activity. On the other hand, alterations of the basic terminus of the amide side chains at the ortho-position of the 2-phenyl group led to varying effects on antibacterial activity. Compounds **5a_4_**, **5a_3_**, **5a_2_** and **5b_4_** showed better antibacterial activity than Compounds **5a_6_**, **5a_7_** and **5b_6_** against *S. aureus* and *B. subtilis*, indicating that rigid cyclic amino group at 2-phenyl is suitable for the anti-Gram-positive bacteria activity. On the contrary, for *E. coli*, Compounds **5a_6_**, **5a_7_** and **5b_6_** exhibited stronger activity than the compounds with rigid cyclic amino substituents(**5a_2_**, **5a_3_**, **5a_4_**, **5b_4_**), suggesting that the flexible chain amino group at 2-phenyl can enhance antibacterial activity against *E. coli.* All of these results demonstrated the importance of the length and flexibility of the amide side chain for antibacterial activity.

In order to further determinate the antibacterial effect of the compounds, the minimum inhibitory concentration (MIC) values against the above-mentioned bacteria strains were measured by a broth dilution method [38,39,40]. The MIC value is a measure to test the antibacterial activity of a compound and is defined as the lowest concentration of antibacterial agent that inhibits visible growth. Ampicillin and gentamycin were used as positive controls in the assay. The MIC values are summarized in Table 2.

The results of MIC values correspond to that obtained by the agar diffusion method as a whole. Some compounds displayed good antibacterial activity against *S. aureus*, and Compound **5a_4_** showed the best inhibition against *S. aureus* with a MIC of 64 μg/mL, which is better than Compound **1**. A few compounds exhibited moderate activity against *E. coli*, and Compound **5a_7_** had the best activity against *E. coli* with a MIC of 128 μg/mL. However, all the compounds exhibited weak activity against *B. subtilis*, *P. aeruginosa* and MRSA; their MICs were more than 256 μg/mL. Unfortunately, all of these compounds exhibited less potent activity than the standard ones against the above-mentioned strains.

### 2.3. MTT-Based Cytotoxicity Studies

The *in vitro* cytotoxicity of Compounds **5a_4_** and **5a_7_**, which showed the best antibacterial activity, were further examined in mouse macrophage cell lines (RAW 264.7) using the MTT colorimetric assay [40,41]. As shown in Table 3, Compounds **5a_4_** and **5a_7_** showed weak cytotoxicity in the RAW 264.7 cells with IC_50_ values of 98.2 μg/mL and 56.8 μg/mL, respectively, which are similar to the cytotoxicities of ampicillin and gentamycin. The low cytotoxicities for mouse macrophage cells of Compounds **5a_4_** and **5a_7_** exhibited their good safety profiles, which indicate that they could be further developed as antibacterial agents for infectious diseases against *S. aureus* and *E. coil*, respectively.

## 3. Experimental Section

### 3.1. General

All reagents and solvents were of analytical grade and used without further purification. All reactions were monitored by TLC on 0.2 mm-thick silica gel GF254 pre-coated plates. All flash column chromatography were performed with silica gel (200–300 mesh) purchased from Qingdao Haiyang Chemical Co. Ltd., Qingdao, China); ^1^H-NMR and ^13^C-NMR spectra were recorded using TMS as the internal standard in CDCl_3_ or DMSO-*d*_6_ with a Bruker BioSpin GmbH spectrometer (Bruker BioSpin, Switzerland) at 400 MHz and 101 MHz, respectively; high resolution mass spectra (HRMS) were recorded on the Shimadzu LCMS-IT-TOF spectrometer (Shimadzu, Tokyo, Japan); the purity of the synthesized compounds was confirmed to be higher than 95% through analytical HPLC (Shimadzu, Tokyo, Japan) performed with a dual pump Shimadzu LC-20AB system (Shimadzu, Tokyo, Japan) equipped with an Ultimate XB-C18 column (4.6 × 250 mm, 5 μm) (Welch, New York, NY USA) and eluted with methanol/water (40:60–70:30) containing 0.1% TFA at a flow rate of 0.5 mL/min.

### 3.2. Chemistry

#### 3.2.1. Preparation of 2-(2-Nitrophenyl)quinoline-4-carboxylic Acid (**1**)

Method A: An equimolar mixture of aniline (1.9 g, 20 mmol) and 2-nitrobenzaldehyde (3.0 g, 20 mmol) in ethanol (30 mL) was refluxed for 1 h; pyruvic acid (2.6 g, 30 mmol) and trifluoroacetic acid (0.1 mL) were then added to the reaction mass and further refluxed for 12 h. Reaction completion was monitored by using TLC. The reaction mixture was poured into ice water (60 mL) slowly with vigorous stirring. The solid product was filtered and was added to the aqueous K_2_CO_3_; the solution was adjusted to basic, then filtered. The filtrate was acidified with diluted HCl to pH 1–2, and the mixture was extracted with three 40-mL portions of CH_2_Cl_2_, The combined organic phase was washed with 40 mL water, dried over anhydrous sodium sulfate and concentrated under reduced pressure. The crude product was purified by using flash column chromatography to give Compound **1** in a 23% yield.

Method B: A mixture of 2-nitrobenzaldehyde (3.0 g, 20 mmol) and pyruvic acid (3.5 g, 39.8 mmol) was stirred for 15 min; the solid was dissolved. Then, 30 mL acetic acid were added, and the mixture was stirred at 100 °C for 30 min. After aniline (3.7 g, 40 mmol) was added, the mixture was refluxed for 8 h. Then, the reaction mixture was cooled to room temperature and poured into ice water (60 mL); then, aqueous NaOH was added to make the solution basic. The mixture was extracted with three 40-mL portions of CH_2_Cl_2_; the water phase was acidified with diluted HCl to pH 1–2, and the precipitated crude product was filtered. A white solid was obtained in a yield of 68% after recrystallization in ethanol. ^1^H-NMR (400 MHz, DMSO*-d*_6_): δ 14.09 (s, 1H), 8.72 (d, *J* = 8.5 Hz, 1H), 8.19 (s, 1H), 8.09 (d, *J* = 7.9 Hz, 1H), 8.03 (d, *J* = 8.2 Hz, 1H), 7.96 (d, *J* = 7.3 Hz, 1H), 7.88 (t, *J* = 7.5 Hz, 2H), 7.78 (t, *J* = 7.6 Hz, 2H).

#### 3.2.2. Preparation of *N*-(3-(Dimethylamino)propyl)-2-(2-nitrophenyl)quinoline-4-carboxamide (**2**)

A mixture of Compound **1** (1.2 g, 4.1 mmol) and SOCl_2_ (15 mL) was stirred under reflux for 3 h. After completion of the reaction, the excess of SOCl_2_ was distilled off. The obtained yellow residue was added dropwise to the mixture of *N*,*N*-dimethylpropylamine (0.5 g, 4.9 mmol), triethylamine(1.6 mL) in CH_2_Cl_2_ (30 mL) at 0 °C, then stirred at room temperature for 10 h. The precipitated solid was collected through filtration; the crude product was purified by using flash column chromatography with CH_2_Cl_2_ elution to afford Compound **2** as a pale yellow liquid in an 86% yield. ^1^H-NMR (400 MHz, CDCl_3_): δ 8.43 (s, 1H), 8.32 (d, *J* = 8.4 Hz, 1H), 8.09 (d, *J* = 8.5 Hz, 1H), 8.00 (d, *J* = 8.1 Hz, 1H), 7.75 (t, *J* = 8.2 Hz, 1H), 7.69 (d, *J* = 4.0 Hz, 2H), 7.62–7.55 (m, 3H), 3.58 (q, *J* = 4.0 Hz, 2H), 2.50 (t, *J* = 6.1 Hz, 2H), 2.20 (s, 6H), 1.84–1.75 (m, 2H).

#### 3.2.3. Preparation of 2-(2-Aminophenyl)-*N*-(3-(dimethylamino)propyl)quinoline-4-carboxamide (**3**)

The mixture of Compound **2** (1.0 g, 2.6 mmol), 80% hydrazine hydrate (1.5 mL) in the presence of 10% Pd/C (0.3 g) and isopropanol (25 mL) was heated under reflux for 2 h, and TLC analysis indicated reaction completion; Pd/C was removed by filtration. After concentration *in vacuo*, 25 mL water were added to the residue; the precipitate was filtered off; the crude product **3** was directly used in the next reaction without purification.

#### 3.2.4. General Preparation of Compounds **4a**–**4b** (GP1)

A solution of the appropriate acid halide (3.9 mmol) in CH_2_Cl_2_ (10 mL) was slowly added to a well-stirred mixture of Compound **3** (0.9 g, 2.6 mmol) and anhydrous potassium carbonate (0.6 g, 4.0 mmol) in CH_2_Cl_2_ (25 mL) at 0 °C. After completing the addition, the reaction was allowed to warm up to room temperature and then stirred overnight. After concentration *in vacuo*, 30 mL water were added to the residue; the precipitate formed was filtered off. Because of the poor solubility of Compound **4**, the crude product was directly used in the next reaction without purification.

#### 3.2.5. General Preparation of Compounds **5a**–**5b** (GP2)

Appropriate amine (2.0 mL) was added to a stirred refluxing suspension of the chloride Compound **4** (0.5 mmol) in MeOH (25 mL). The mixture was stirred under reflux for 10 h, then cooled to room temperature. After concentration *in vacuo*, 50 mL water were added to the residue. The mixture was extracted with three 30-mL portions of CH_2_Cl_2_. The combined organic phase was washed with 40 mL water, dried over anhydrous sodium sulfate and concentrated under reduced pressure. The crude product was purified by using flash column chromatography to afford Compounds **5a**–**5b**.

*N-(3-(Dimethylamino)propyl)-2-(2-(3-(4-methylpiperazin-1-yl)propanamido)phenyl)quinoline-4-carboxamide* (**5a_1_**)

Compound **4a** was reacted with N-methyl piperazine according to GP2 to give the desired product **5a_1_**. After column chromatography with CH_2_Cl_2_/MeOH/Et_3_N (50:1:0.1) elution, a white solid was obtained in a 64% yield. mp. 182.3–184.2 °C. ^1^H-NMR (400 MHz, CDCl_3_): δ 12.40 (s, 1H), 8.53 (d, *J* = 8.3 Hz, 1H), 8.38 (t, *J* = 8.0 Hz, 2H), 8.10 (d, *J* = 8.4 Hz, 1H), 7.92 (s, 1H), 7.81 (t, *J* = 9.2 Hz, 2H), 7.64 (t, *J* = 8.0 Hz, 1H), 7.46 (t, *J* = 7.9 Hz, 1H), 7.23 (t, *J* = 8.0 Hz, 1H), 3.69 (q, *J* = 4.0 Hz, 2H), 2.81 (t, *J* = 7.0 Hz, 2H), 2.64 (t, *J* = 7.1 Hz, 2H), 2.59–2.54 (m, 2H), 2.47 (s, 4H), 2.24 (d, *J* = 5.4 Hz, 6H), 2.19 (s, 3H), 2.07 (s, 4H), 1.91–1.84 (m, 2H). ^13^C-NMR (101 MHz, CDCl_3_): δ 170.41, 166.76, 157.68, 146.99, 143.79, 137.86, 130.66, 130.62, 129.34, 128.83, 127.69, 125.83, 125.67, 123.69, 123.16, 122.47, 118.60, 59.06, 54.79 (2C), 53.94, 52.66 (2C), 45.75, 45.31 (2C), 40.53, 35.65, 25.23. Purity: 99.4% by HPLC. HRMS (ESI): Calcd. for [M + H]^+^ (C_29_H_39_N_6_O_2_) requires *m*/*z* 503.3129, found 503.3106.

*N-(3-(Dimethylamino)propyl)-2-(2-(3-(pyrrolidin-1-yl)propanamido)phenyl)quinoline-4-carboxamide* (**5a_2_**)

Compound **4a** was reacted with pyrrolidine according to GP2 to give the desired product **5a_2_**. After column chromatography with CH_2_Cl_2_/MeOH/Et_3_N (100:1:0.1) elution, a yellow solid was obtained in a 65% yield. mp. 176.7–178.1 °C. ^1^H-NMR (400 MHz, CDCl_3_): δ 12.57 (s, 1H), 8.56 (d, *J* = 8.3 Hz, 1H), 8.41 (s, 1H), 8.35 (d, *J* = 8.4 Hz, 1H), 8.11 (d, *J* = 8.4 Hz, 1H), 7.89 (s, 1H), 7.82–7.74 (m, 2H), 7.62 (t, *J* = 7.6 Hz, 1H), 7.43 (t, *J* = 7.8 Hz, 1H), 7.19 (t, *J* = 7.6 Hz, 1H), 3.67 (dd, *J* = 11.4, 5.8 Hz, 2H), 2.90 (t, *J* = 7.3 Hz, 2H), 2.69 (t, *J* = 7.3 Hz, 2H), 2.54–2.48 (m, 6H), 2.19 (s, 6H), 1.87–1.80 (m, 2H), 1.66 (s, 4H). ^13^C-NMR (101 MHz, CDCl_3_): δ 170.29, 166.79, 157.62, 146.92, 143.79, 138.03, 130.67, 130.60, 129.23, 128.93, 127.68, 125.77, 125.58, 123.55, 123.08, 122.25, 118.45, 59.16, 53.92 (2C), 51.84, 45.40 (2C), 40.64, 37.50, 25.33, 23.36 (2C). Purity: 98.8% by HPLC. HRMS (ESI): Calcd. for [M + H]^+^ (C_28_H_36_N_5_O_2_) requires *m*/*z* 474.2864, found 474.2837.

*N-(3-(Dimethylamino)propyl)-2-(2-(3-(diethylamino)propanamido)phenyl)quinoline-4-carboxamide* (**5a_3_**)

Compound **4a** was reacted with diethylamine according to GP2 to give the desired product **5a_3_**. After column chromatography with CH_2_Cl_2_/MeOH/Et_3_N (100:1:0.1) elution, a pale yellow solid was obtained in a 59% yield. mp. 177.2–178.4 °C. ^1^H-NMR (400 MHz, CDCl_3_): δ 12.60 (s, 1H), 8.56 (d, *J* = 8.3 Hz, 1H), 8.41 (s, 1H), 8.35 (d, *J* = 8.4 Hz, 1H), 8.10 (d, *J* = 8.4 Hz, 1H), 7.91 (s, 1H), 7.79 (t, *J* = 7.7 Hz, 2H), 7.62 (t, *J* = 7.7 Hz, 1H), 7.44 (t, *J* = 7.8 Hz, 1H), 7.19 (t, *J* = 7.6 Hz, 1H), 3.67 (q, *J* = 5.4 Hz, 2H), 2.93 (t, *J* = 7.2 Hz, 2H), 2.63 (t, *J* = 7.2 Hz, 2H), 2.58–2.50 (m, 6H), 2.20 (s, 6H), 1.87–1.80 (m, 2H), 0.97 (t, *J* = 7.1 Hz, 6H). ^13^C-NMR (101 MHz, CDCl_3_): δ 170.62, 166.85, 157.54, 146.76, 143.71, 138.07, 130.59 (2C), 129.21, 128.79, 127.63, 125.57, 125.28, 123.50, 123.05, 122.12, 118.42, 58.87, 48.66, 46.71 (2C), 45.31 (2C), 40.33, 35.93, 25.45, 11.36 (2C). Purity: 99.7% by HPLC. HRMS (ESI): Calcd. for [M + H]^+^ (C_28_H_38_N_5_O_2_) requires *m*/*z* 476.3020, found 476.2994.

*N-(3-(Dimethylamino)propyl)-2-(2-(3-(piperidin-1-yl)propanamido)phenyl)quinoline-4-carboxamide* (**5a_4_**)

Compound **4a** was reacted with piperidine according to GP2 to give the desired product **5a_4_**. After column chromatography with CH_2_Cl_2_/MeOH/Et_3_N (100:1:0.1) elution, a yellow solid was obtained in a 60% yield. mp. 168.9–170.1 °C. ^1^H-NMR (400 MHz, CDCl_3_): δ 12.52 (s, 1H), 8.51 (d, *J* = 8.3 Hz, 1H), 8.41–8.30 (m, 2H), 8.12 (d, *J* = 8.5 Hz, 1H), 7.90 (s, 1H), 7.83–7.75 (m, 2H), 7.63(t, *J* = 8.0 Hz, 1H), 7.44 (t, *J* = 7.8 Hz, 1H), 7.21 (t, *J* = 7.6 Hz, 1H), 3.67 (q, *J* = 5.6 Hz, 2H), 2.78 (t, *J* = 7.1 Hz, 2H), 2.67 (t, *J* = 7.1 Hz, 2H), 2.51 (t, *J* = 6.0 Hz, 2H), 2.39 (s, 4H), 2.20 (s, 6H), 1.87–1.80 (m, 2H), 1.47–1.39 (m, 4H), 1.36–1.32 (m, 2H). ^13^C-NMR (101 MHz, CDCl_3_): δ 170.56, 166.86, 157.58, 146.94, 143.72, 137.88, 130.62, 130.57, 129.30, 128.94, 127.66, 125.85, 125.56, 123.69, 123.08, 122.45, 118.53, 58.92, 54.58, 54.15 (2C), 45.32 (2C), 40.40, 35.45, 25.52 (2C), 25.40, 24.01. Purity: 99.2% by HPLC. HRMS (ESI): Calcd. for [M + H]^+^ (C_29_H_38_N_5_O_2_) requires *m*/*z* 488.3020, found 488.2995.

*N-(3-(Dimethylamino)propyl)-2-(2-(3-morpholinopropanamido)phenyl)quinoline-4-carboxamide* (**5a_5_**)

Compound **4a** was reacted with morpholine according to GP2 to give the desired product **5a_5_**. After column chromatography with CH_2_Cl_2_/MeOH/Et_3_N (90:1:0.1) elution, a pale yellow solid was obtained in a 59% yield. mp. 188.1–189.4 °C. ^1^H-NMR (400 MHz, CDCl_3_): δ 12.45 (s, 1H), 8.51 (d, *J* = 8.6 Hz, 1H), 8.45 (s, 1H), 8.37 (d, *J* = 8.0 Hz, 1H), 8.10–8.05 (m, 2H), 7.97 (d, *J* = 12.0 Hz, 1H), 7.81 (t, *J* = 8.0 Hz, 1H), 7.64 (t, *J* = 8.0 Hz, 1H), 7.45 (t, *J* = 8.0 Hz, 1H), 3.73–3.69 (m, 2H), 3.52 (s, 4H), 2.98 (s, 2H), 2.81 (s, 2H), 2.67–2.61 (m, 8H), 2.43 (s, 4H), 2.15–2.12 (m, 2H). ^13^C-NMR (101 MHz, CDCl_3_): δ 170.54, 166.83, 157.65, 146.93, 143.75, 137.95, 130.63, 130.59, 129.29, 128.88, 127.66, 125.80, 125.57, 123.66, 123.08, 122.43, 118.49, 66.83 (2C), 58.97, 53.65, 52.79 (2C), 45.40 (2C), 40.29, 35.70, 25.38. Purity: 97.6% by HPLC. HRMS (ESI): Calcd. for [M + H]^+^ (C_28_H_36_N_5_O_3_) requires *m*/*z* 490.2813, found 490.2785.

*N-(3-(Dimethylamino)propyl)-2-(2-(3-((3-(dimethylamino)propyl)amino)propanamido)phenyl)quinoline-4-carboxamide* (**5a_6_**)

Compound **4a** was reacted with 3-dimethyl aminopropyl amine according to GP2 to give the desired product **5a_6_**. After column chromatography with CH_2_Cl_2_/MeOH/Et_3_N (85:2:0.1) elution, a brown solid was obtained in a 55% yield. mp. 171.5–173.4 °C. ^1^H-NMR (400 MHz, CDCl_3_): δ12.97 (s, 1H), 8.59 (d, *J* = 8.2 Hz, 1H), 8.47 (s, 1H), 8.33 (d, *J* = 8.4 Hz, 1H), 8.12 (d, *J* = 8.4 Hz, 1H), 7.96 (s, 1H), 7.88–7.79 (m, 2H), 7.63 (t, *J* = 7.8 Hz, 1H), 7.46 (t, *J* = 7.8 Hz, 1H), 7.23 (t, *J* = 7.8 Hz, 1H), 3.72–3.65 (m, 2H), 3.64 (s, 1H), 3.11 (t, *J* = 5.5 Hz, 2H), 2.97–2.84 (m, 4H), 2.54 (t, *J* = 6.1 Hz, 2H), 2.43 (t, *J* = 6.3 Hz, 2H), 2.25 (s, 6H), 2.22 (s, 6H), 1.90–1.84 (m, 2H), 1.78–1.70 (m, 2H). ^13^C-NMR (101 MHz, CDCl_3_): δ 171.51, 167.96, 158.57, 147.83, 145.03, 139.21, 131.80, 131.74, 130.31, 129.90, 128.77, 126.66, 126.07, 124.60, 124.14, 123.01, 119.45, 58.93, 49.66, 48.35, 46.52 (2C), 45.77 (2C), 45.42, 41.57, 38.77, 26.60, 25.89. Purity: 98.8% by HPLC. HRMS (ESI): Calcd. for [M + H]^+^ (C_29_H_41_N_6_O_2_) requires *m*/*z* 505.3286, found 505.3164.

*N-(3-(Dimethylamino)propyl)-2-(2-(3-((3-(diethylamino)propyl)amino)propanamido)phenyl)quinoline-4-carboxamide* (**5a_7_**)

Compound **4a** was reacted with 3-diethyl aminopropyl amine according to GP2 to give the desired product **5a_7_**. After column chromatography with CH_2_Cl_2_/MeOH/Et_3_N (85:2:0.1) elution, brown oil was obtained in a 54% yield. ^1^H-NMR (400 MHz, CDCl_3_): δ 12.82 (s, 1H), 8.61 (d, *J* = 8.4 Hz, 1H), 8.45 (s, 1H), 8.35 (d, *J* = 8.4 Hz, 1H), 8.12 (d, *J* = 8.4 Hz, 1H), 7.93 (s, 1H), 7.81 (t, *J* = 7.6 Hz, 2H), 7.63 (t, *J* = 7.6 Hz, 1H), 7.46 (t, *J* = 7.8 Hz, 1H), 7.22 (t, *J* = 8.4 Hz, 1H), 3.68 (q, *J* = 5.6 Hz, 2H), 3.03 (t, *J* = 5.9 Hz, 2H), 2.78 (t, *J* = 4.0 Hz, 2H), 2.70 (t, *J* = 4.0 Hz, 2H), 2.55–2.44 (m, 8H), 2.19 (s, 6H), 1.88–1.82 (m, 2H), 1.67–1.58 (m, 2H), 1.00 (t, *J* = 7.0 Hz, 6H). ^13^C-NMR (101 MHz, CDCl_3_): δ 170.41, 166.86, 157.47, 146.73, 143.93, 138.11, 130.70, 130.64, 129.21, 128.80, 127.67, 125.56, 124.97, 123.50, 123.04, 121.91, 118.35, 59.00, 51.05, 48.56, 46.63 (2C), 45.42 (2C), 45.32, 40.47, 37.67, 26.18, 25.50, 11.30 (2C). Purity: 98.4% by HPLC. HRMS (ESI): Calcd. for [M + H]^+^ (C_31_H_45_N_6_O_2_) requires *m*/*z* 533.3599, found 533.3568.

*N-(3-(Dimethylamino)propyl)-2-(2-(2-(4-methylpiperazin-1-yl)acetamido)phenyl)quinoline-4-carboxamide* (**5b_1_**)

Compound **4b** was reacted with *N*-methyl piperazine according to GP2 to give the desired product **5b_1_**. After column chromatography with CH_2_Cl_2_/MeOH/Et_3_N (50:1:0.1) elution, a yellow solid was obtained in a 67% yield. mp. 185.5–186.8 °C. ^1^HNMR (400 MHz, CDCl_3_): δ 11.74 (s, 1H), 8.55 (d, *J* = 12.0 Hz, 1H), 8.48 (t, *J* = 4.0 Hz, 1H), 8.41 (d, *J* = 8.3 Hz, 1H), 8.27 (d, *J* = 8.4 Hz, 1H), 7.85- 7.73 (m, 2H), 7.66–7.57 (m, 2H), 7.43 (d, *J* = 7.4 Hz, 1H), 7.19 (t, *J* = 6.5 Hz, 1H), 3.68–3.62 (m, 2H), 3.11 (s, 2H), 2.50 (t, *J* = 4.0 Hz, 2H), 2.42 (s, 4H), 2.20 (s, 7H), 1.98 (s, 6H), 1.87–1.79 (m, 2H). ^13^C-NMR (101 MHz, CDCl_3_): δ 169.28, 166.87, 157.53, 147.50, 143.71, 136.42, 130.36, 130.12(2C), 129.75, 128.08, 127.66, 125.66, 123.91, 123.30, 122.46, 119.06, 63.30, 58.74, 53.84 (2C), 53.25 (2C), 45.58, 45.26 (2C), 40.21, 25.35. Purity: 96.9% by HPLC. HRMS (ESI): Calcd. for [M + H]^+^ (C_28_H_37_N_6_O_2_) requires *m*/*z* 489.2973, found 489.3036.

*N-(3-(Dimethylamino)propyl)-2-(2-(2-(pyrrolidin-1-yl)acetamido)phenyl)quinoline-4-carboxamide* (**5b_2_**)

Compound **4b** was reacted with pyrrolidine according to GP2 to give the desired product **5b_2_**. After column chromatography with CH_2_Cl_2_/MeOH/Et_3_N (100:1:0.1) elution, a yellow solid was obtained in a 66% yield. mp. 180.1–180.9 °C. ^1^H-NMR (400 MHz, CDCl_3_): δ 12.32 (s,1H), 8.60 (d, *J* = 8.3 Hz, 1H), 8.38 (t, *J* = 8.4 Hz, 2H), 8.20 (d, *J* = 8.4 Hz, 1H), 7.82 (s, 1H), 7.76 (t, *J* = 8.4 Hz, 1H), 7.66 (d, *J* = 7.8 Hz, 1H), 7.61 (t, *J* = 8.4 Hz, 1H), 7.44 (t, *J* = 8.4 Hz, 1H), 7.20 (t, *J* = 8.4 Hz, 1H), 3.70–3.62 (m, 2H), 3.27 (s, 2H), 2.53–2.45 (m, 6H), 2.21 (s, 6H), 1.87–1.79 (m, 2H), 1.51 (s, 4H). ^13^C-NMR (101 MHz, CDCl_3_): δ 170.27, 166.92, 157.40, 147.35, 143.62, 137.01, 130.18, 129.92, 129.49, 129.41, 127.53, 127.38, 125.42, 123.65, 123.00, 122.51, 118.67, 61.47, 58.98, 54.50 (2C), 45.35 (2C), 40.43, 25.41, 23.84 (2C). Purity: 97.0% by HPLC. HRMS (ESI): Calcd. for [M + H]^+^ (C_27_H_34_N_5_O_2_) requires *m*/*z* 460.2707, found 460.2682.

*N-(3-(Dimethylamino)propyl)-2-(2-(2-(diethylamino)acetamido)phenyl)quinoline-4-carboxamide* (**5b_3_**)

Compound **4b** was reacted with diethylamine according to GP2 to give the desired product **5b_3_**. After column chromatography with CH_2_Cl_2_/MeOH/Et_3_N (100:1:0.1) elution, a yellow solid was obtained in a 63% yield. mp. 175.4–176.8 °C. ^1^H-NMR (400 MHz, CDCl_3_): δ 12.24 (s, 1H), 8.59 (d, *J* = 8.3 Hz, 1H), 8.45–8.35 (m, 2H), 8.19 (d, *J* = 8.4 Hz, 1H), 7.80(s, 1H), 7.78 (t, *J* = 7.8 Hz, 1H), 7.67–7.58 (m, 2H), 7.46 (t, *J* = 7.8 Hz, 1H), 7.21 (t, *J* = 7.6 Hz, 1H), 3.66 (q, *J* = 4.0 Hz, 2H), 3.13 (s, 2H), 2.54–2.44 (m, 6H), 2.19 (s, 6H), 1.86–1.80 (m, 2H), 0.78 (t, *J* = 8.0 Hz, 6H). ^13^C-NMR (101 MHz, CDCl_3_): δ 170.34, 166.96, 157.43, 147.38, 143.66, 137.00, 130.24, 129.97, 129.55, 129.44, 127.58, 127.44, 125.46, 123.72, 123.02, 122.56, 118.72, 61.48, 59.06, 54.54 (2C), 45.40 (2C), 40.53, 25.35, 23.86 (2C). Purity: 98.2% by HPLC. HRMS (ESI): Calcd. for [M + H]^+^ (C_27_H_36_N_5_O_2_) requires *m*/*z* 462.2865, found 462.2750.

*N-(3-(Dimethylamino)propyl)-2-(2-(2-(piperidin-1-yl)acetamido)phenyl)quinoline-4-carboxamide* (**5b_4_**)

Compound **4b** was reacted with piperidine according to GP2 to give the desired product **5b_4_**. After column chromatography with CH_2_Cl_2_/MeOH/Et_3_N (100:1:0.1) elution, a yellow solid was obtained in a 65% yield. mp. 173.2–174.3 °C. ^1^H-NMR (400 MHz, CDCl_3_): δ 11.79 (s, 1H), 8.56 (d, *J* = 8.3 Hz, 1H), 8.39 (d, *J* = 8.4 Hz, 2H), 8.30 (d, *J* = 8.4 Hz, 1H), 7.81–7.76 (m, 2H), 7.63 (t, *J* = 8.4 Hz, 2H), 7.45 (t, *J* = 8.0 Hz, 1H), 7.20 (t, *J* = 7.5 Hz, 1H), 3.67 (t, *J* = 8.0 Hz, 2H), 3.07 (s, 2H), 2.52 (t, *J* = 8.0 Hz, 2H), 2.34 (s, 4H), 2.20 (s, 6H), 1.88–1.80 (m, 2H), 1.21 (s, 6H). ^13^C-NMR (101 MHz, CDCl_3_): δ 169.65, 166.88, 157.45, 147.41, 143.74, 136.91, 130.36, 130.17, 129.94, 129.86, 127.87, 127.24, 125.58, 123.86, 123.23, 122.55, 119.00, 61.69, 59.10, 54.58 (2C), 45.39 (2C), 40.63, 25.48, 23.87 (2C), 23.26. Purity: 98.9% by HPLC. HRMS (ESI): Calcd. for [M + H]^+^ (C_28_H_36_N_5_O_2_) requires *m*/*z* 474.2864, found 474.2839.

*N-(3-(Dimethylamino)propyl)-2-(2-(2-morpholinoacetamido)phenyl)quinoline-4-carboxamide* (**5b_5_**)

Compound **4b** was reacted with morpholine according to GP2 to give the desired product **5b_5_**. After column chromatography with CH_2_Cl_2_/MeOH/Et_3_N (90:1:0.1) elution, a white solid was obtained in a 68% yield. mp. 171.3–172.7 °C. ^1^H-NMR (400 MHz, CDCl_3_): δ 11.91 (s, 1H), 8.54 (d, *J* = 8.3 Hz, 1H), 8.44 (s, 1H), 8.37 (d, *J* = 8.4 Hz, 1H), 8.25 (d, *J* = 8.4 Hz, 1H), 7.83–7.76 (m, 2H), 7.66–7.59 (m, 2H), 7.44 (t, *J* = 7.8 Hz, 1H), 7.20 (t, *J* = 7.5 Hz, 1H), 3.65 (q, *J* = 5.4 Hz, 2H), 3.34 (d, *J* = 3.9 Hz, 4H), 3.12 (s, 2H), 2.49 (t, *J* = 6.0 Hz, 2H), 2.44–2.37 (m, 4H), 2.19 (s, 6H), 1.87–1.80 (m, 2H). ^13^C-NMR (101 MHz, CDCl_3_): δ 168.82, 166.77, 157.55, 147.36, 143.82, 136.55, 130.31, 130.21, 129.68, 129.61, 127.77, 127.37, 125.66, 123.96, 123.17, 122.58, 118.96, 66.08 (2C), 63.98, 58.96, 53.70 (2C), 45.30 (2C), 40.47, 25.29. Purity: 96.6% by HPLC. HRMS (ESI): Calcd. for [M + H]^+^ (C_27_H_34_N_5_O_3_) requires *m*/*z* 476.2656, found 476.2995.

*N-(3-(Dimethylamino)propyl)-2-(2-(2-((3-(dimethylamino)propyl)amino)acetamido)phenyl)quinoline-4-carboxamide* (**5b_6_**)

Compound **4b** was reacted with 3-dimethyl aminopropyl amine according to GP2 to give the desired product **5b_6_**. After column chromatography with CH_2_Cl_2_/MeOH/Et_3_N (85:2:0.1) elution, yellow oil was obtained in a 56% yield. ^1^H-NMR (400 MHz, CDCl_3_): δ 12.55 (s, 1H), 8.62 (d, *J* = 7.8 Hz, 1H), 8.50 (s, 1H), 8.34 (d, *J* = 8.7 Hz, 1H), 8.21 (d, *J* = 9.1 Hz, 1H), 7.87 (s, 1H), 7.83–7.78 (m, 1H), 7.75 (d, *J* = 7.9 Hz, 1H), 7.63 (t, *J* = 6.9 Hz, 1H), 7.47 (t, *J* = 7.9 Hz, 1H), 7.24 (t, *J* = 8.0 Hz, 1H), 3.68 (d, *J* = 4.4 Hz, 2H), 3.47 (d, *J* = 2.3 Hz, 2H), 2.60 (d, *J* = 4.0 Hz, 4H), 2.28–2. 22 (m, 8H), 2.19 (d, *J* = 2.1 Hz, 6H), 1.90 (t, *J* = 4.0 Hz, 2H), 1.48–1.41 (m, 2H). ^13^C-NMR (101 MHz, CDCl_3_): δ 170.19, 166.93, 157.48, 147.45, 143.67, 136.68, 130.28, 130.10, 129.75, 129.42, 127.66, 127.40, 125.60, 123.91, 123.09, 122.52, 118.73, 60.87, 59.26, 58.94, 46.50, 45.95 (2C), 45.30 (2C), 40.38, 25.63, 25.26. Purity: 95.6% by HPLC. HRMS (ESI): Calcd. for [M + H]^+^ (C_28_H_39_N_6_O_2_) requires *m*/*z* 491.3135, found 491.3217.

### 3.3. Antibacterial Studies

#### 3.3.1. Screening for Antibacterial Activity by the Agar Diffusion Method

The *in vitro* antibacterial activities of newly-synthesized Compounds **5a_1_**–**5a_7_** and **5b_1_**–**5b_6_** were evaluated for their antibacterial activities against *S. aureus* (ATCC29213), *B. subtilis* (ATCC6633), *E. coli* (ATCC25922), *P. aeruginosa* (ATCC9027) and MRSA (ATCC43300) bacterial strains by the agar diffusion method [36]. Fifteen to twenty milliliters of agar media were poured into each petri dish. Agar-containing plates were dried by placing in a laminar air flow at 37.2 °C for 1 h.

One hundred milliliters of 0.5 McFarland standard of bacterial suspension were inoculated on the agar media and spread on the whole surface with a sterile cotton bud. Using a sterile cork borer, 5-mm wells were made on the seeded agar plates, and 50 mL of test compound at different concentrations (50 and 100 μg/mL) were transferred in to the wells. The plates were prepared in triplicate for each compound. A control was also prepared for the plates in the same way using solvent DMSO. The plates were maintained at 37 ± 2 °C for 24 h. Activities were determined by measuring the diameter of inhibition zone (mm). Ampicillin and gentamycin were used as the standards.

#### 3.3.2. Minimum Inhibitory Concentration Determination

The MIC values were measured by the broth dilution method [40,41]. Five hundred microliters of a stock solution (10.24 mg/mL) of each tested compound in dimethyl sulfoxide (DMSO) were prepared and then diluted with Mueller-Hinton broth to 1024 μg/mL. The strains were grown briefly at 37 °C in Mueller-Hinton media. After 5 h of bacterial growth, the bacterial culture was diluted to obtain a concentration of 5 × 10^5^ cells/mL. Then, 150 μL bacterial suspension were added to each well of the flat-bottomed 96-well tissue culture plate. Two-fold serial dilutions were carried out from the fist well to the tenth well; the final concentrations of the compounds ranged from 1–512 μg/mL; and excess media (150 μL) were discarded from the last well. The plates were incubated at 37 °C for 24 h in an electro-heating standing temperature cultivator and were read visually. The MIC of the sample showing no turbidity was recorded as the lowest concentration of compound that inhibited bacterial growth completely. Each assay was run in triplicate.

#### 3.3.3. Cytotoxicity Assay

The active Compounds **5a_4_** and **5a_7_** were further examined for cytotoxicity in mouse macrophage cell lines (RAW 264.7) [41]. The cells were cultured in DMEM medium supplemented with 10% fetal bovine serum, 2 mM L-glutamine and 100 units/mL penicillin/streptomycin. Cell cultures were maintained in a humidified atmosphere of 5% CO_2_ at 37 °C. Cells were passaged at pre-confluent densities using a solution containing 0.25% trypsin and 0.5 mM EDTA. Cells were seeded at 1 × 10^4^ cell/well in a 96-well plate for 12 h with 10% FBS, 1% penicillin and streptomycin and 1% l-glutamine, resulting in 80% confluency. Each dose was prepared in 1% FBS medium by 1000× dilution of the drug, which was prepared in DMSO solution to ensure a DMSO concentration less than 0.1%. After 72 h of exposure, the viability was assessed on the basis of cellular conversion of MTT into a formazan product with DMSO for 45 min. The amount of formazan was measured using a microculture plate reader with a test wavelength of 570 nm. Results were expressed as the mean ± SD of three independent experiments.

^1^H-NMR, ^13^C-NMR, HRMS and HPLC spectra of the target compounds are shown in the Supplementary Materials.

## 4. Conclusions

In summary, thirteen novel 2-phenyl-quinoline-4-carboxylic acid derivatives were synthesized using aniline, 2-nitrobenzaldehyde and pyruvic acid as starting materials. The structures of the new compounds were elucidated by using ^1^H-NMR, ^13^C-NMR and HRMS, and their purities were determined to be above 95% by using HPLC. The newly-synthesized compounds were investigated for their *in vitro* antibacterial activities by the agar diffusion method and a broth dilution method. The results of antibacterial evaluation suggested that some compounds displayed moderate antibacterial activity against *S. aureus*, and Compound **5a_4_** showed the best inhibition against *S. aureus* with a MIC of 64 μg/mL. A few compounds exhibited weak activity against *E. coli*, and Compound **5a_7_** had the best activity against *E. coli* with a MIC of 128 μg/mL. The cytotoxic assay exhibited that the active Compound **5a_4_** showed weak cytotoxicity. These findings indicate the potential usefulness of **5a_4_** in drug development. Moreover, some structure-activity relationships of 2-phenyl-quinoline-4-carboxylic acid derivatives were determined. The length and flexibility of the amide side chain at the ortho-position of the 2-phenyl group had a significant effect on antibacterial activity. The compound with longer side chains showed stronger activity than the compound with shorter side chains. Moreover, rigid cyclic amino group at 2-phenyl is suitable for the antibacterial activity against *S. aureus* and *B. subtilis*, and the introduction of flexible chain amino group at 2-phenyl can enhance antibacterial activity against *E. coli.* All of these results will be useful in the future to guide the design and modification of new candidate 2-phenyl-quinoline-4-carboxylic acid analogues as antibacterial agents.

## Figures and Tables

**Table 1:** Antibacterial activities of the synthesized compounds determined by the agar diffusion method.

	Zone of Inhibition in mm (Mean ± SD) *n* = 3											
Compound	LogP	*B. subtilis*		*S. aureus*		*E. coli*		*P. aeruginosa*		*MRSA*	
Concentration (μg/mL)		50	100	50	100	50	100	50	100	50	100
**5a_1_**	0.32	1 ± 0.2	2 ± 0.5	3 ± 0.2	5 ± 0.4	2 ± 0.1	2 ± 0.5	1 ± 0.1	1 ± 0.2	3 ± 0.2	3 ± 0.4
**5a_2_**	1.69	2 ± 0.3	2 ± 0.2	15 ± 0.5	17 ± 0.1	4 ± 0.2	5 ± 0.5	2 ± 0.5	3 ± 0.4	3 ± 0.1	6 ± 0.3
**5a_3_**	1.05	10 ± 0.1	11 ± 0.5	14 ± 0.2	16 ± 0.5	3 ± 0.2	3 ± 0.4	2 ± 0.2	2 ± 0.5	4 ± 0.3	5 ± 0.4
**5a_4_**	2.26	12 ± 0.3	13 ± 0.2	18 ± 0.2	20 ± 0.1	2 ± 0.5	3 ± 0.2	0	0	5 ± 0.5	6 ± 0.2
**5a_5_**	0.60	6 ± 0.2	7 ± 0.4	7 ± 0.1	8 ± 0.5	0	0	3 ± 0.1	5 ± 0.1	2 ± 0.4	3 ± 0.5
**5a_6_**	0.87	0	0	0	0	10 ± 0.2	13 ± 0.1	1 ± 0.2	2 ± 0.4	0	0
**5a_7_**	1.94	0	0	1 ± 0.5	2 ± 0.1	12 ± 0.5	14 ± 0.1	0	0	0	0
**5b_1_**	0.72	1 ± 0.1	1 ± 0.2	2 ± 0.1	3 ± 0.5	0	0	0	0	0	0
**5b_2_**	1.75	3 ± 0.3	4 ± 0.2	4 ± 0.2	7 ± 0.1	2 ± 0.3	3 ± 0.4	0	0	0	0
**5b_3_**	1.20	3 ± 0.5	3 ± 0.1	4 ± 0.1	6 ± 0.3	4 ± 0.2	6 ± 0.4	1 ± 0.2	2 ± 0.1	0	0
**5b_4_**	2.32	10 ± 0.1	12 ± 0.5	8 ± 0.5	10 ± 0.1	3 ± 0.3	5 ± 0.1	0	0	5 ± 0.2	7 ± 0.4
**5b_5_**	1.00	1 ± 0.2	3 ± 0.3	4 ± 0.5	5 ± 0.4	0	0	0	0	0	0
**5b_6_**	0	0	0	0	0	10 ± 0.5	11 ± 0.2	3 ± 0.1	4 ± 0.3	0	0
**1**	3.54	2 ± 0.5	3 ± 0.1	3 ± 0.5	5 ± 0.1	3 ± 0.1	4 ± 0.3	1 ± 0.4	2 ± 0.2	0	0
**Control ^a^**		0	0	0	0	0	0	0	0	0	0
**Ampicillin**		32 ± 0.3	- ^b^	25 + 0.4	-	15 ± 0.4	-	12 ± 0.2	-	1 ± 0.4	1 ± 0.1
**Gentamycin**		10 ± 0.5	-	14 ± 0.3	-	17 ± 0.1	-	20 ± 0.4	-	0	0

^a^ Dimethyl sulfoxide; ^b^ not determined.

**Table 2:** The minimum inhibitory concentration (MIC) values of the synthesized compounds against bacteria.

Compound	MIC (μg/ mL)				
	*B. subtilis*	*S. aureus*	*E. coli*	*P. aeruginosa*	*MRSA*
**5a_1_**	>512	>512	>512	>512	>512
**5a_2_**	>512	128	512	>512	512
**5a_3_**	256	128	>512	>512	>512
**5a_4_**	256	64	>512	-^a^	512
**5a_5_**	512	256	-	512	>512
**5a_6_**	-	-	256	>512	-
**5a_7_**	-	>512	128	-	-
**5b_1_**	>512	>512	-	-	-
**5b_2_**	>512	512	>512	-	-
**5b_3_**	>512	512	512	>512	-
**5b_4_**	256	128	>512	-	512
**5b_5_**	>512	>512	-	-	-
**5b_6_**	-	-	256	>512	-
**1**	>512	512	>512	>512	-
**Ampicillin**	16	16	64	64	512
**Gentamycin**	64	16	16	8	-

^a^ Not determined.

**Table 3:** IC_50_ (μg/mL) values of the compounds against mouse macrophage cell lines (RAW 264.7 ^a^).

Compound	IC_50_^b^ (μg/mL)
**5a_4_**	98.24 ± 0.65
**5a_7_**	56.83 ± 0.32
**Ampicillin**	107.58± 0.47
**Gentamycin**	42.19 ± 0.19

^a^ RAW 264.7 monolayers were used as an *in vitro* model of cervicovaginal epithelium for testing the cytotoxicity of the new compounds; ^b^ IC_50_ is defined as the concentration at which 50% of growth is inhibited.

## Supplementary Materials

Supplementary data (^1^H-NMR, ^13^C-NMR, HRMS and HPLC spectra) associated with this article are available at: http://www.mdpi.com/1420-3049/21/3/340/s1.
