# Supplementary material for: Design, Synthesis and Antibacterial Evaluation of Some New 2-Phenyl-quinoline-4-carboxylic Acid Derivatives
Source: Molecules. 2016 Mar 10;21(3):340. doi: 10.3390/molecules21030340 (PMC6273947; doi:10.3390/molecules21030340)
Supplement: Supplementary file 1 [file molecules-21-00340-s001.pdf]

Supplementary Materials: Design, Synthesis, and Antibacterial Evaluation of Some New 2-Phenyl-Quinoline-4-Carboxylic Acid Derivatives

Xiaoqin Wang, Xiaoyang Xie, Yuanhong Cai, Xiaolan Yang, Jiayu Li, Yinghan Li, Wenna Chen and Minghua He

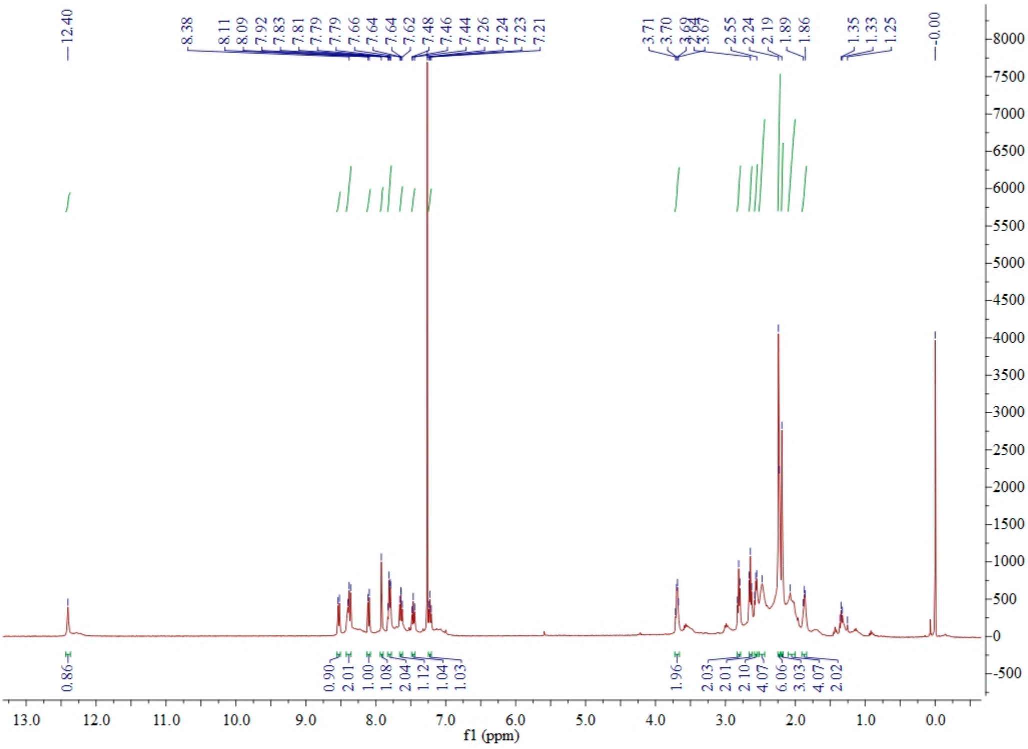

Figure S1. ^{1}H-NMR Spectrum of compound **5a**1.

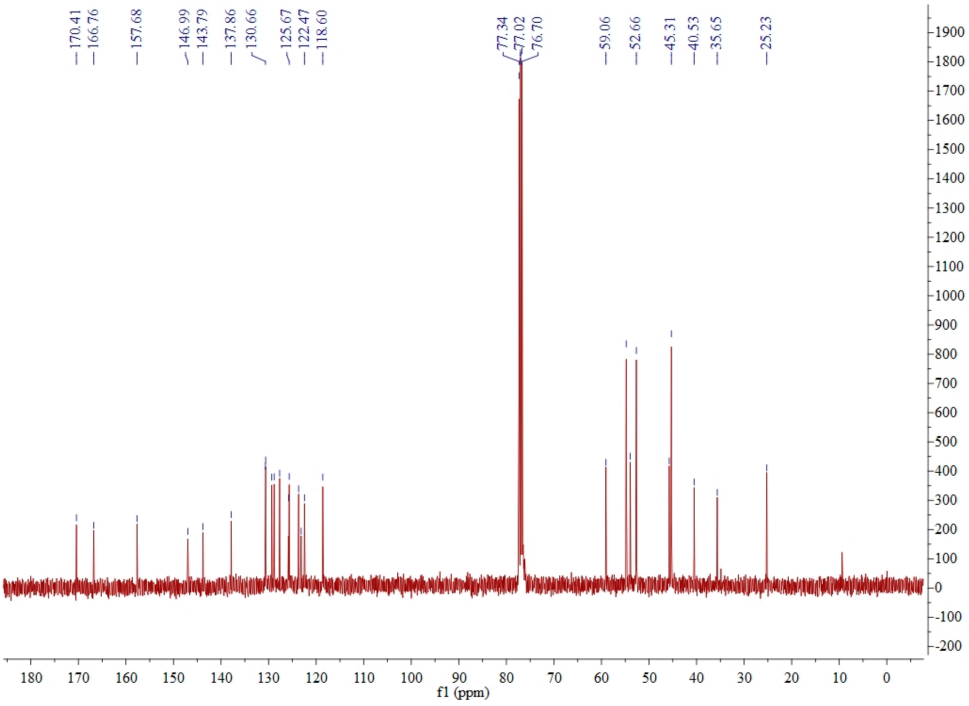

Figure S2. ^{13}C-NMR Spectrum of compound **5a**1.

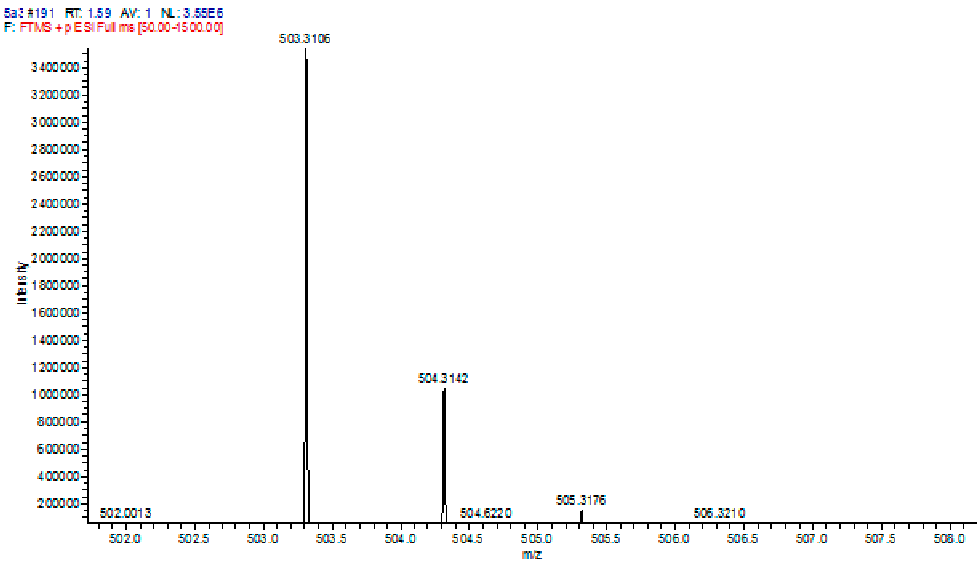

Idx	Formula	RDB	Delta mmu
1	$C_{29}H_{39}O_2N_6$	13.5	-2.541

Calculated 503.3129 Found 503.3106

Figure S3. HRMS Spectrum of compound **5a1**.

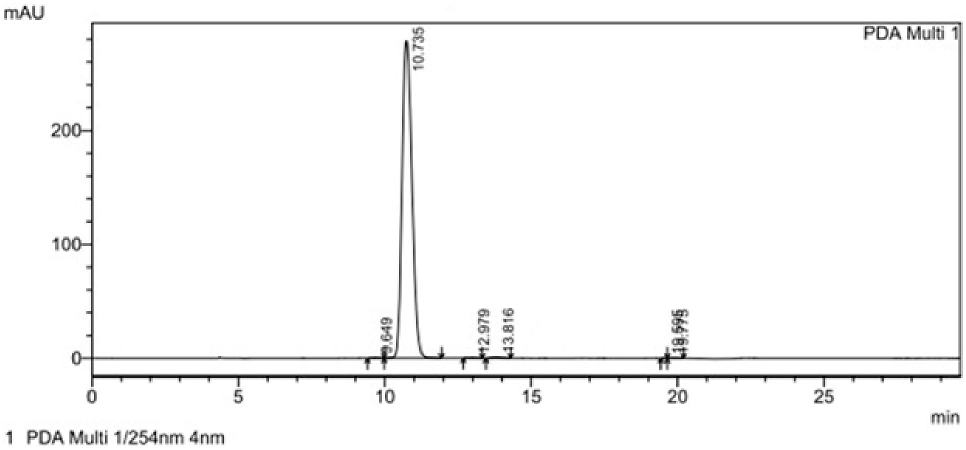

PeakTable

PDA Ch1 254nm 4nm

Peak#	Ret. Time	Area	Height	Area %	Height %
1	9.649	5706	327	0.088	0.117
2	10.735	6443787	278615	99.377	99.286
3	12.979	5285	269	0.082	0.096
4	13.816	23472	1039	0.362	0.370
5	19.595	1096	138	0.017	0.049
6	19.775	4842	229	0.075	0.082
Total		6484188	280617	100.000	100.000

Figure S4. HPLC Spectrum of compound **5a1**.

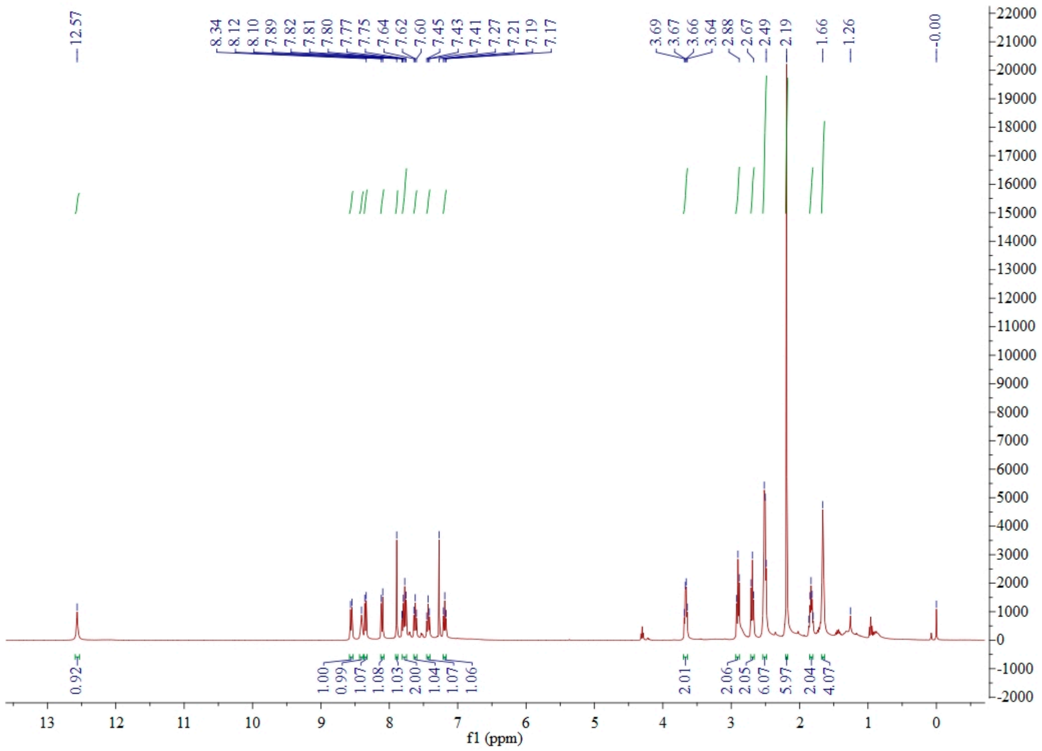

Figure S5. ^{1}H-NMR Spectrum of compound **5a**2.

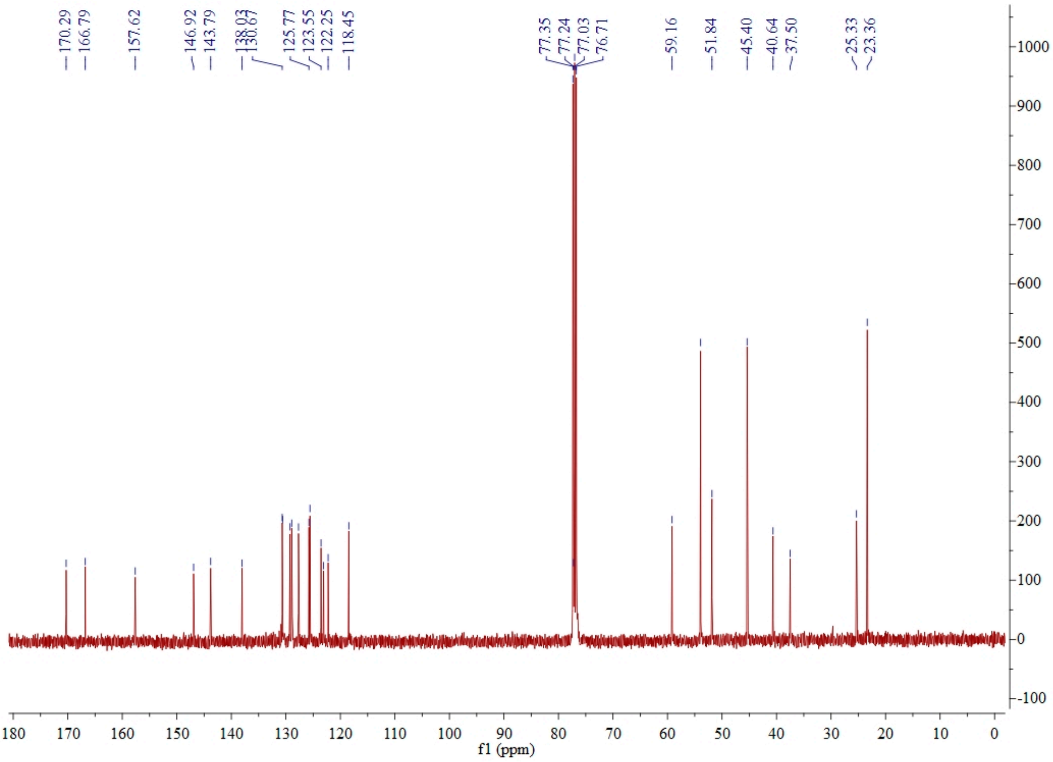

Figure S6. ^{13}C-NMR Spectrum of compound **5a**2.

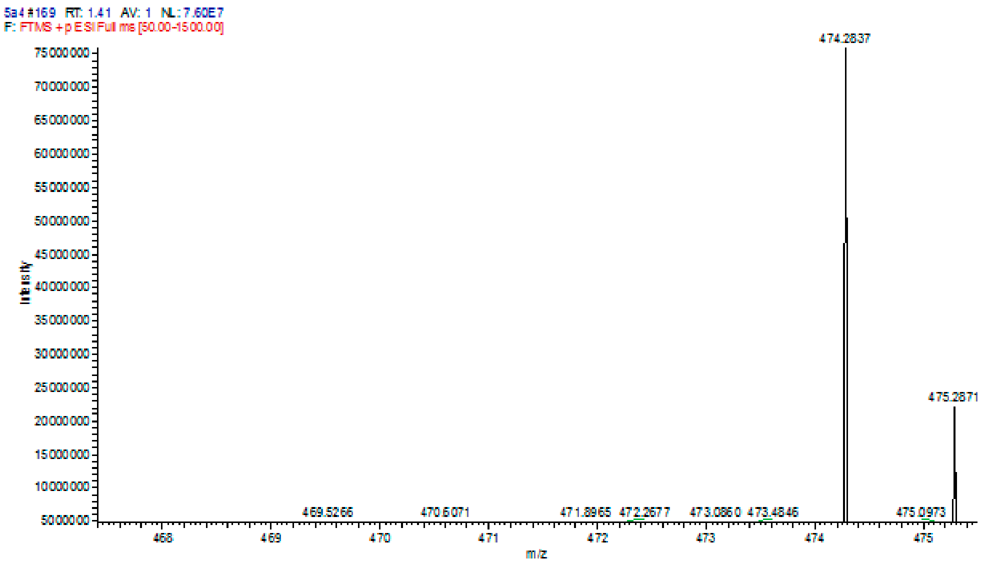

Idx	Formula	RDB	Delta mmu
1	$C_{28}H_{36}O_2N_5$	13.5	-2.632

Calculated 474.2864 Found 474.2837

Figure S7. HRMS Spectrum of compound **5a**2.

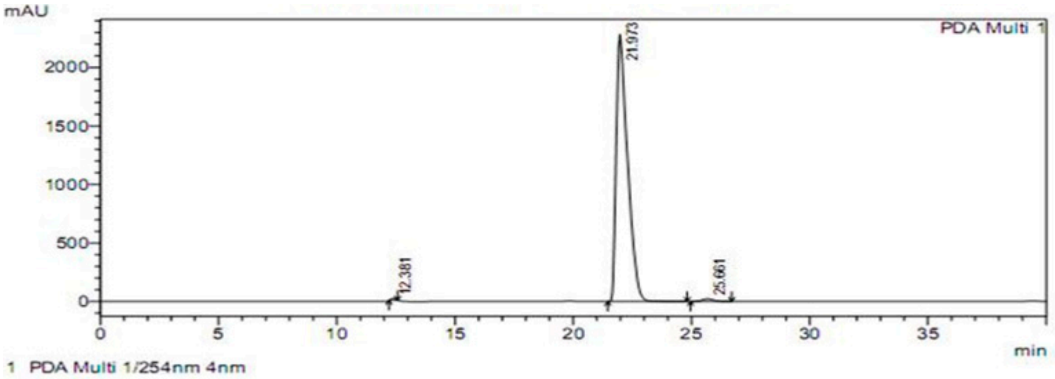

PeakTable

PDA Ch1 254nm 4nm

Peak#	Ret. Time	Area	Height	Area %	Height %
1	12.381	196282	14877	0.256	0.641
2	21.973	75648089	2284692	98.770	98.441
3	25.661	745739	21295	0.974	0.918
Total		76590110	2320864	100.000	100.000

Figure S8. HPLC Spectrum of compound **5a**2.

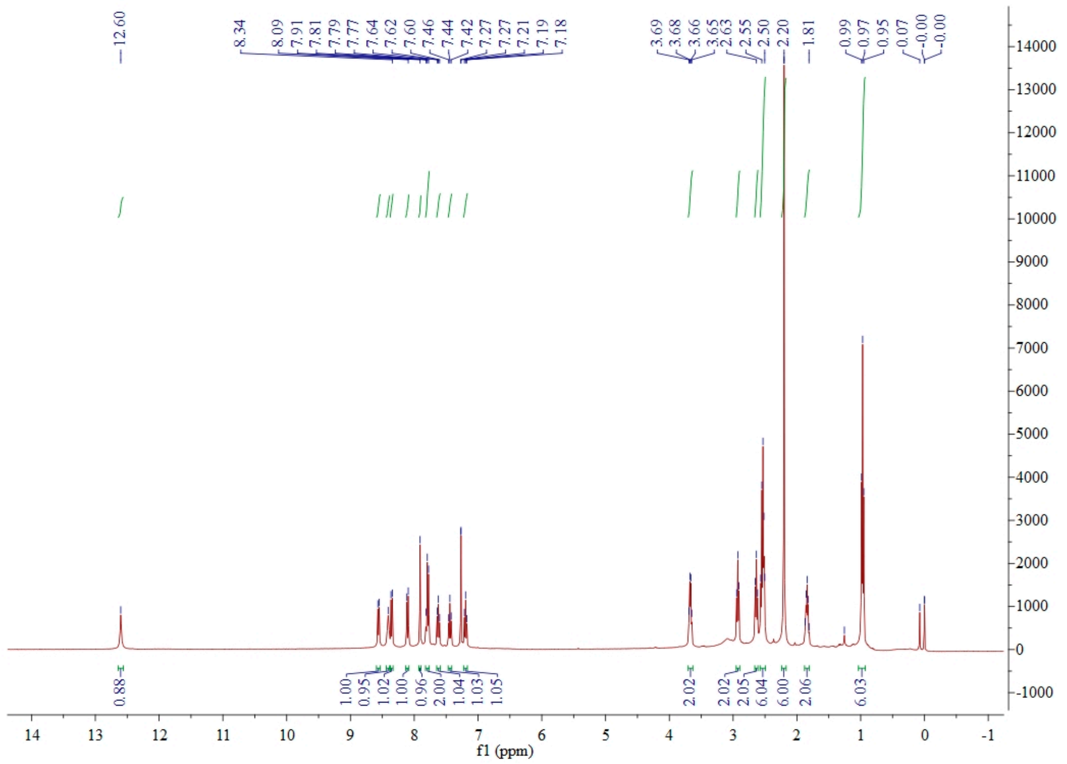

Figure S9. ¹H-NMR Spectrum of compound **5a**3.

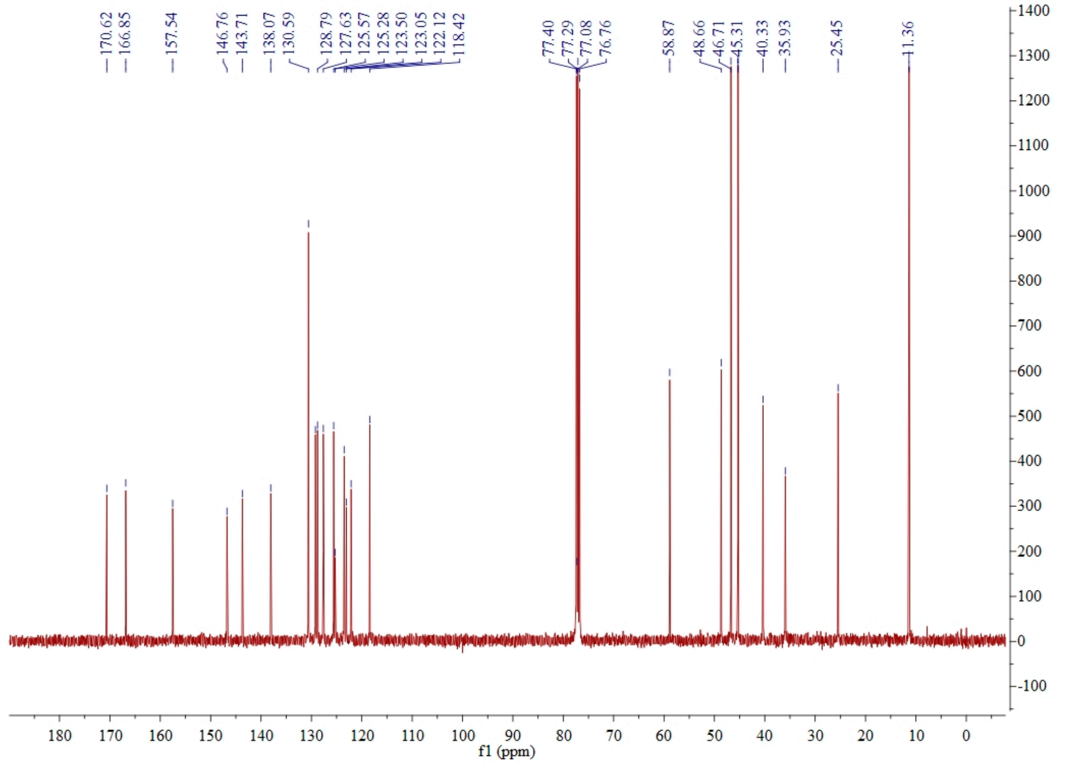

Figure S10. ¹³C-NMR Spectrum of compound **5a**3.

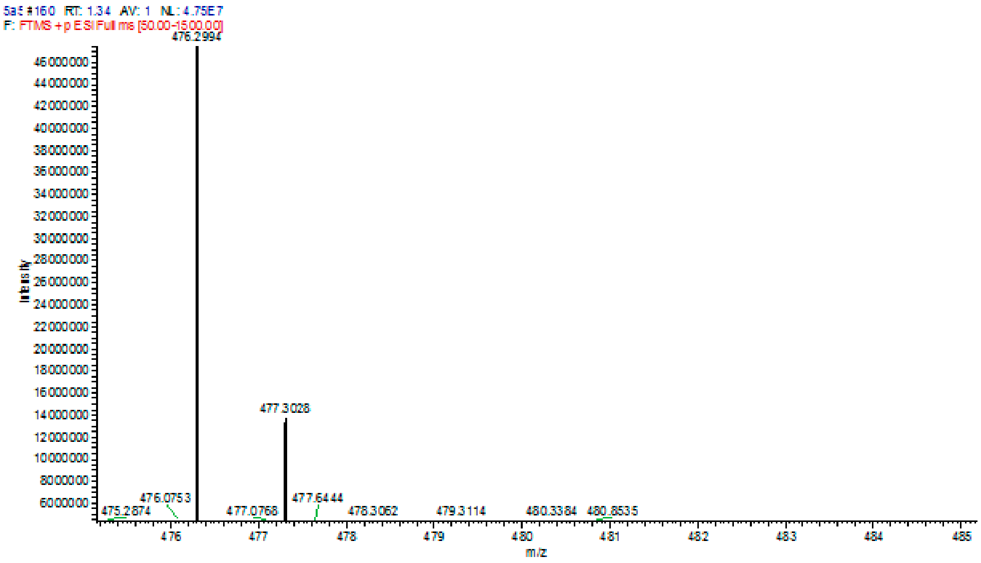

Idx	Formula	RDB	Delta mmu
1	$C_{28}H_{38}O_2N_5$	12.5	-2.652

Calculated 476.3020 Found 476.2994

Figure S11. HRMS Spectrum of compound **5a**3.

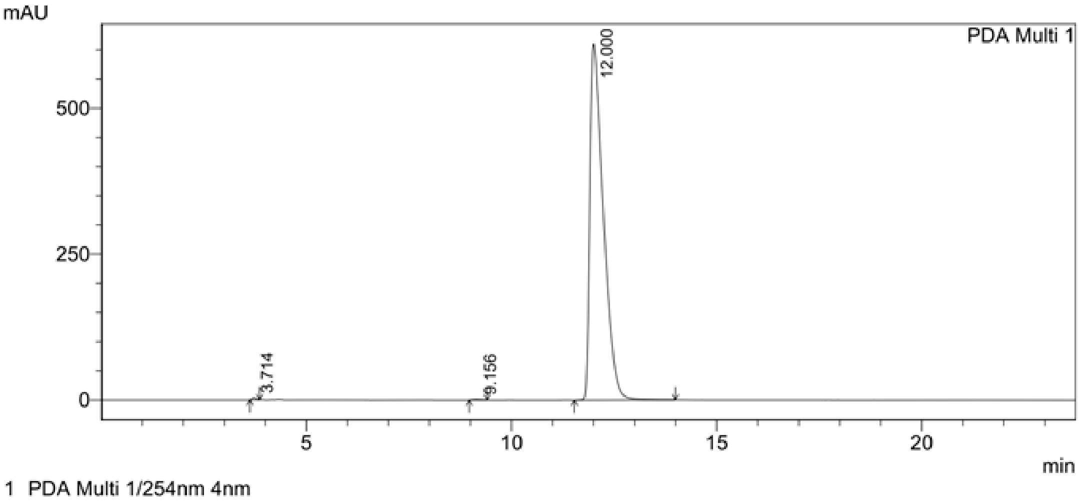

PeakTable

PDA Ch1 254nm 4nm

Peak#	Ret. Time	Area	Height	Area %	Height %
1	3.714	23334	3522	0.167	0.573
2	9.156	13924	1050	0.099	0.171
3	12.000	13969692	610445	99.734	99.257
Total		14006950	615017	100.000	100.000

Figure S12. HPLC Spectrum of compound **5a**3.

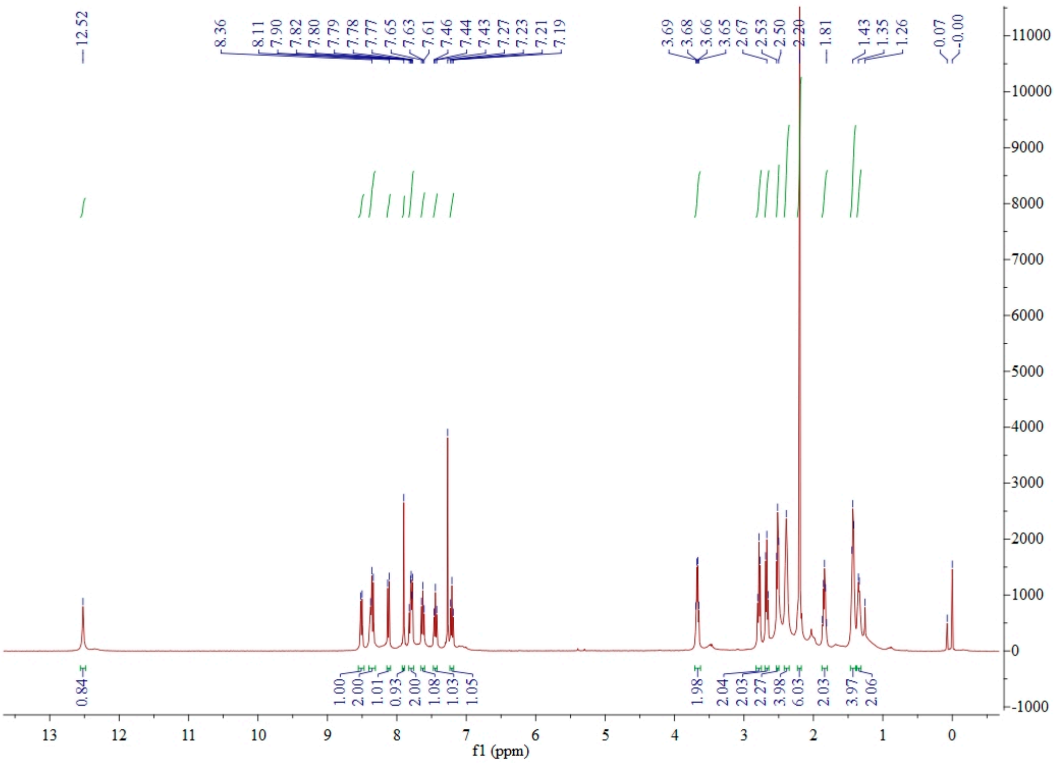

Figure S13. ^{1}H-NMR Spectrum of compound **5a**$_4$.

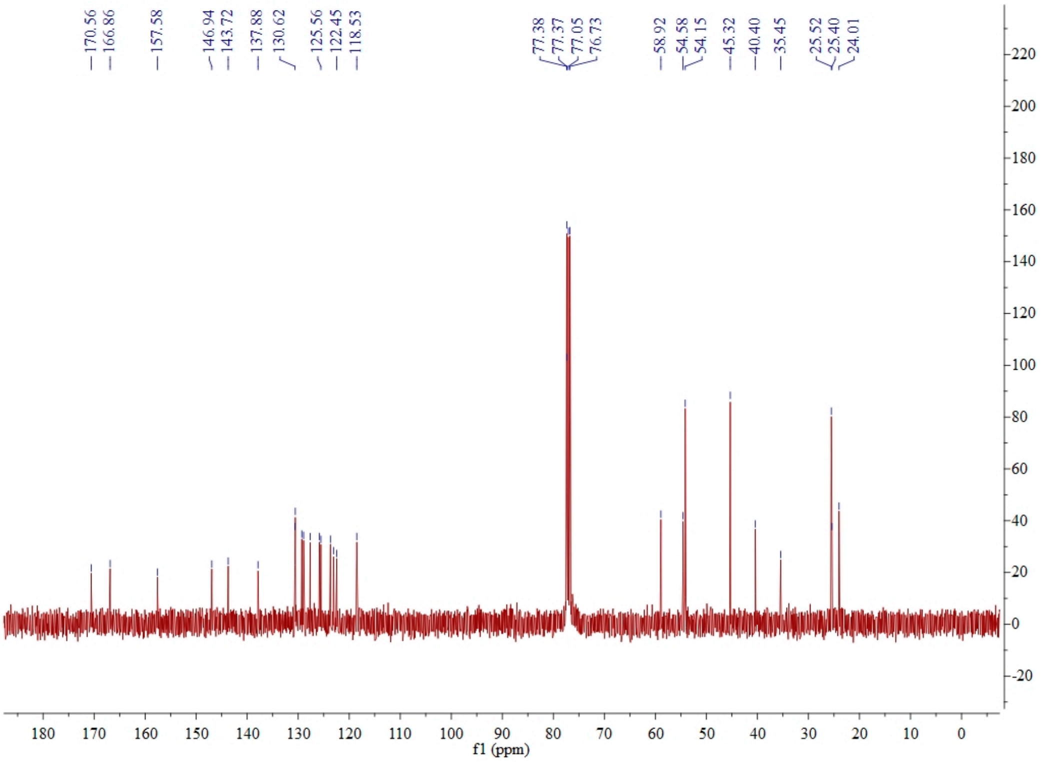

Figure S14. ^{13}C-NMR Spectrum of compound **5a**$_4$.

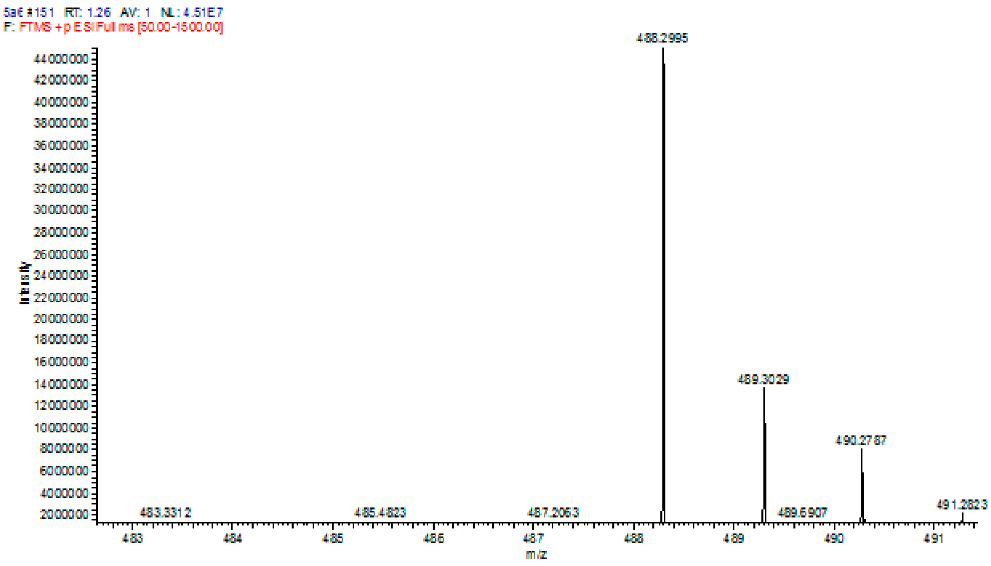

Idx	Formula	RDB	Delta mmu
1	$C_{29}H_{38}O_2N_5$	13.5	-2.472

Calculated 488.3020 Found 488.2995

Figure S15. HRMS Spectrum of compound **5a**4.

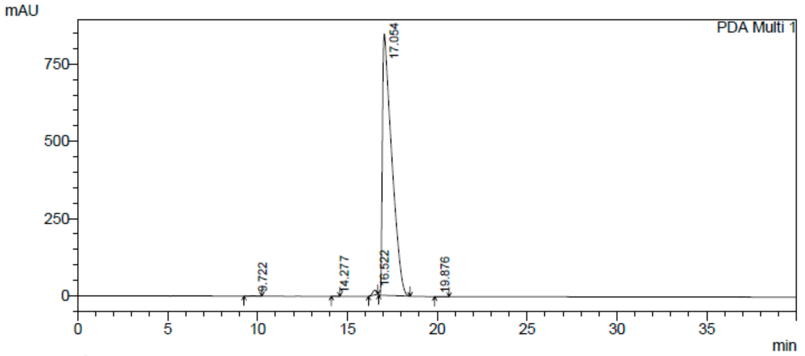

PeakTable

PDA Ch1 254nm 4nm

Peak#	Ret. Time	Area	Height	Area %	Height %
1	9.722	20263	1065	0.066	0.123
2	14.277	7824	559	0.025	0.065
3	16.522	229029	15481	0.744	1.794
4	17.054	30519574	845975	99.163	98.015
5	19.876	446	23	0.001	0.003
Total		30777136	863104	100.000	100.000

Figure S16. HPLC Spectrum of compound **5a**4.

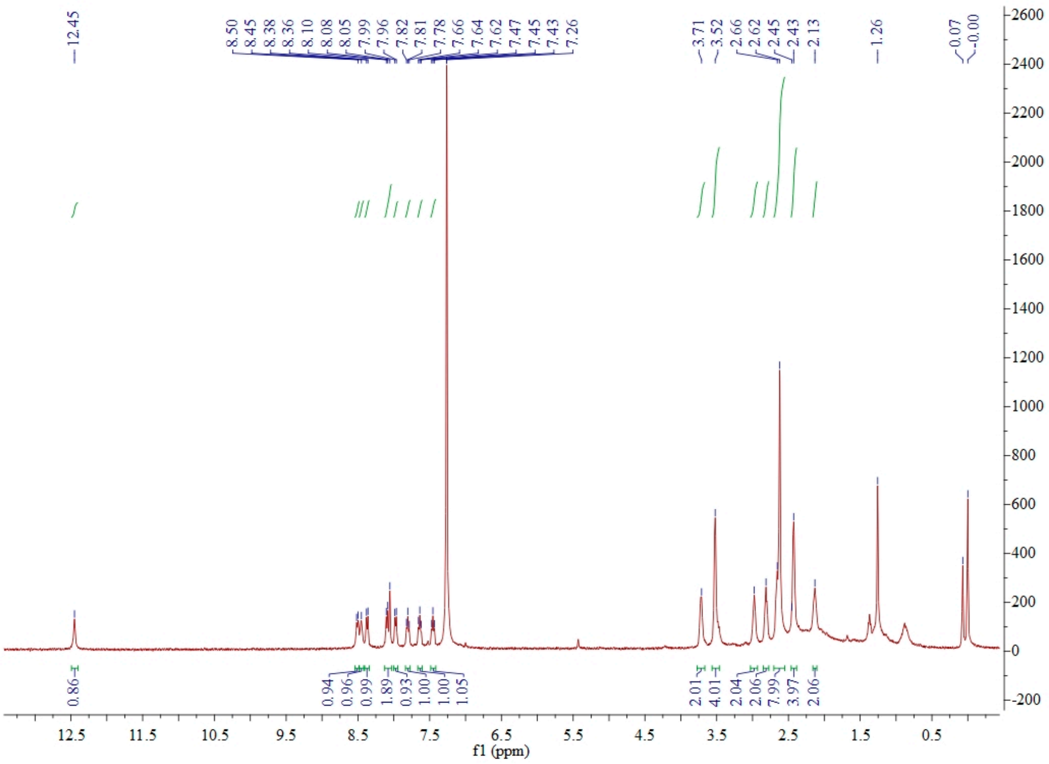

Figure S17. ^{1}H-NMR Spectrum of compound **5a**5.

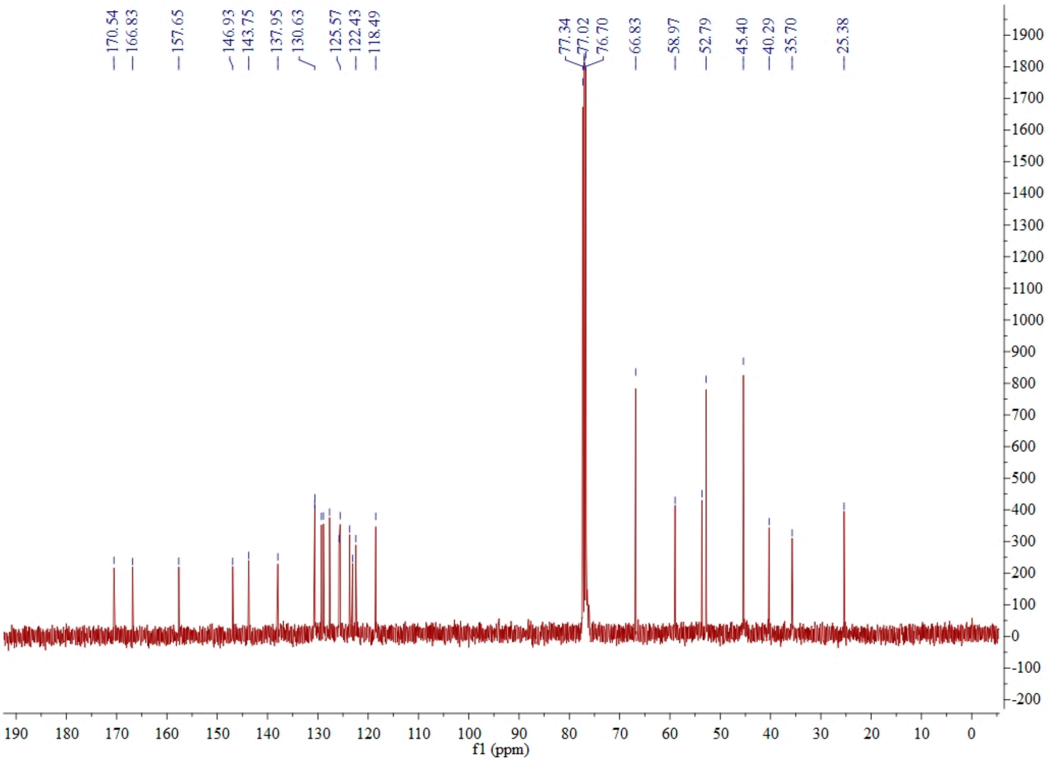

Figure S18. ^{13}C-NMR Spectrum of compound **5a**5.

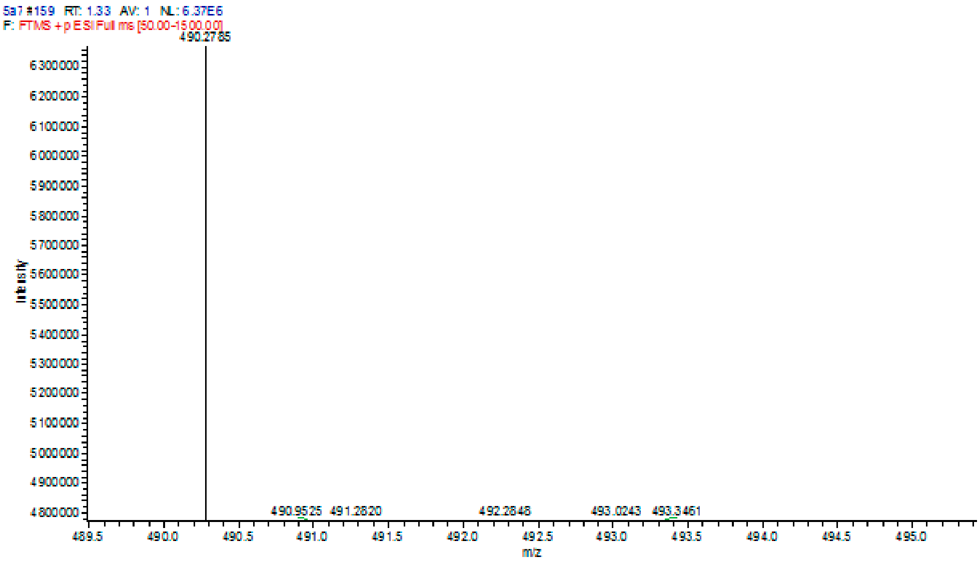

Idx	Formula	RDB	Delta mmu
1	$C_{28}H_{36}O_3N_5$	13.5	-2.736

Calculated 490.2813 Found 490.2785

Figure S19. HRMS Spectrum of compound **5a**5.

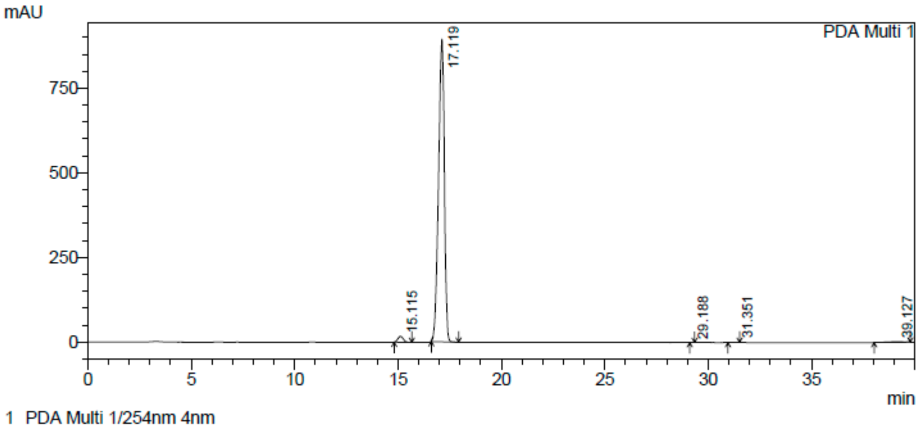

PeakTable

PDA Ch1 254nm 4nm

Peak#	Ret. Time	Area	Height	Area %	Height %
1	15.115	317080	17821	1.766	1.951
2	17.119	17530804	893596	97.615	97.803
3	29.188	393	71	0.002	0.008
4	31.351	8292	404	0.046	0.044
5	39.127	102600	1773	0.571	0.194
Total		17959169	913665	100.000	100.000

Figure S20. HPLC Spectrum of compound **5a**5.

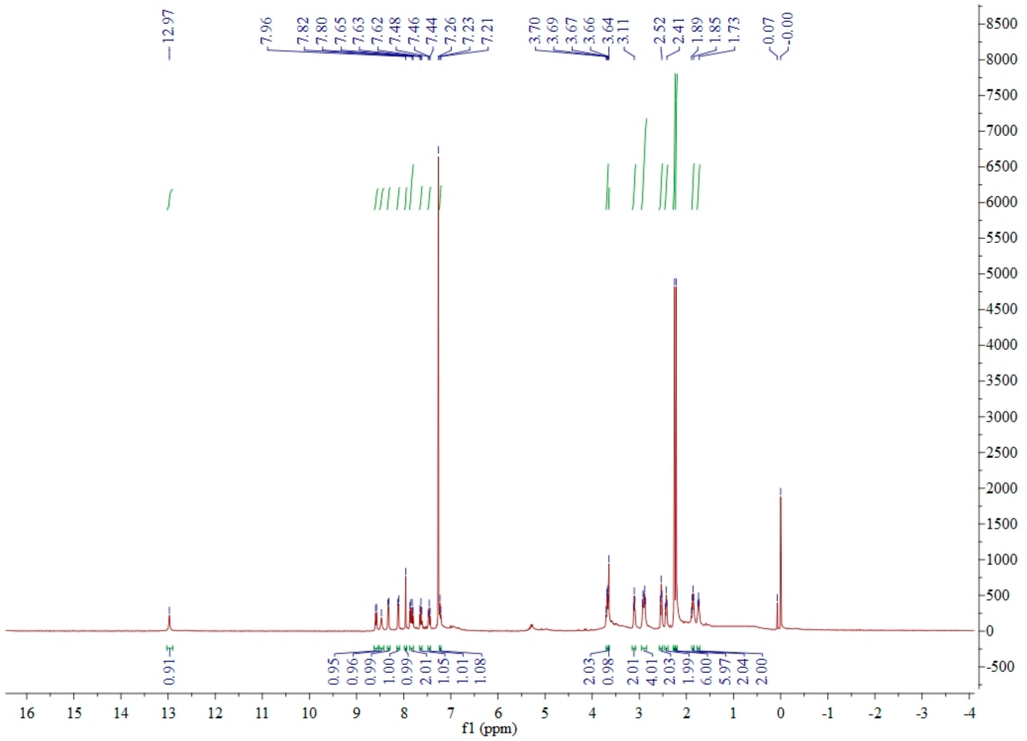

Figure S21. ¹H-NMR Spectrum of compound **5a6**.

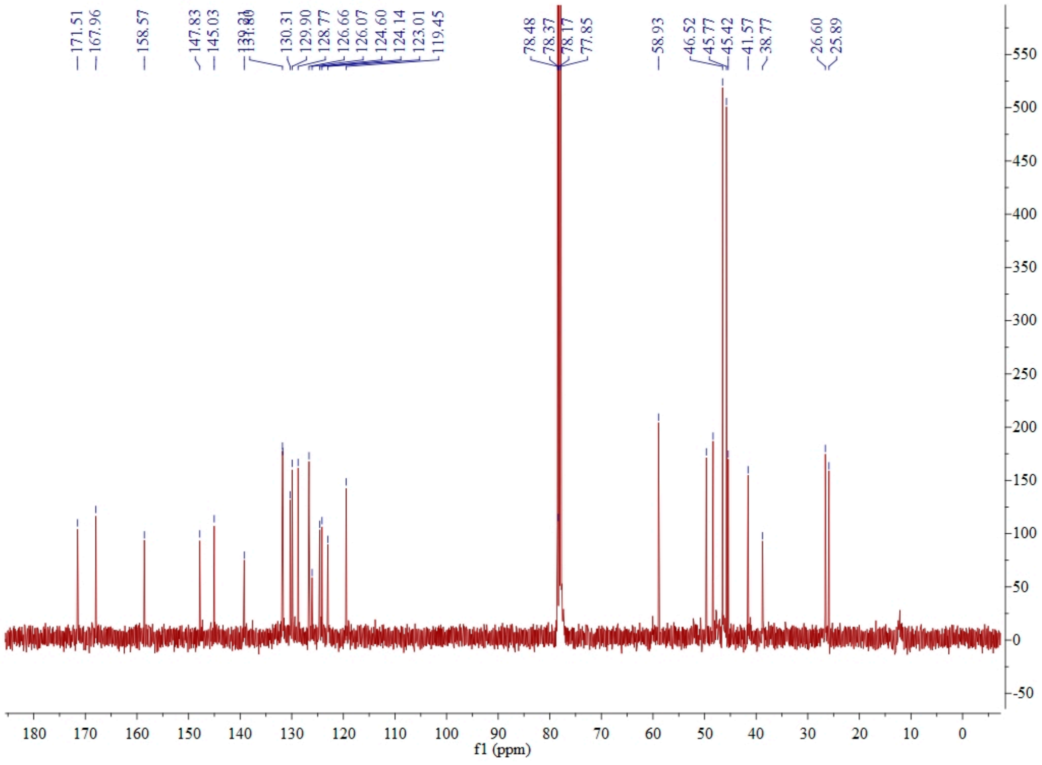

Figure S22. ¹³C-NMR Spectrum of compound **5a6**.

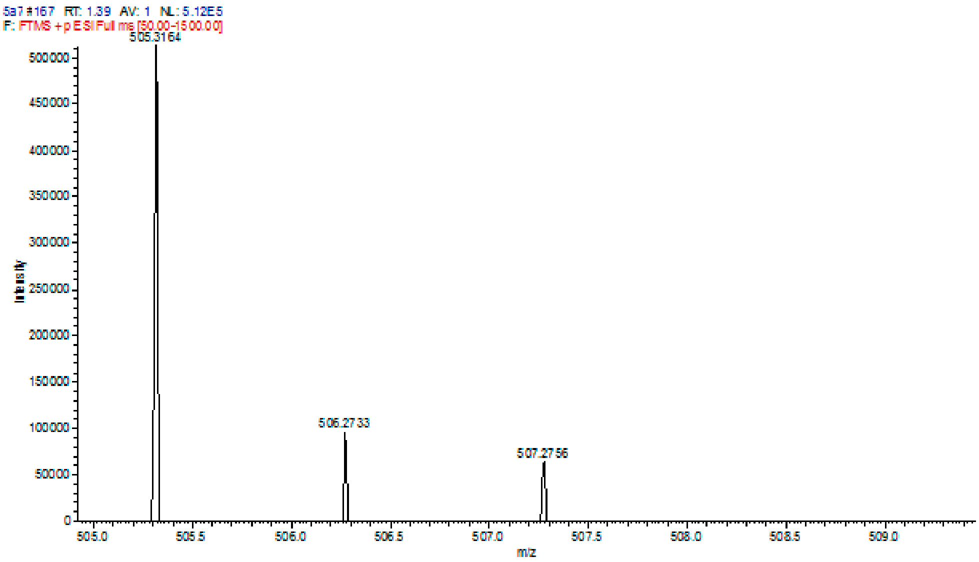

Idx	Formula	RDB	Delta mmu
1	$C_{29}H_{41}O_2N_6$	12.5	-12.111

Calculated 505.3286 Found 505.3164

Figure S23. HRMS Spectrum of compound **5a$_6$**.

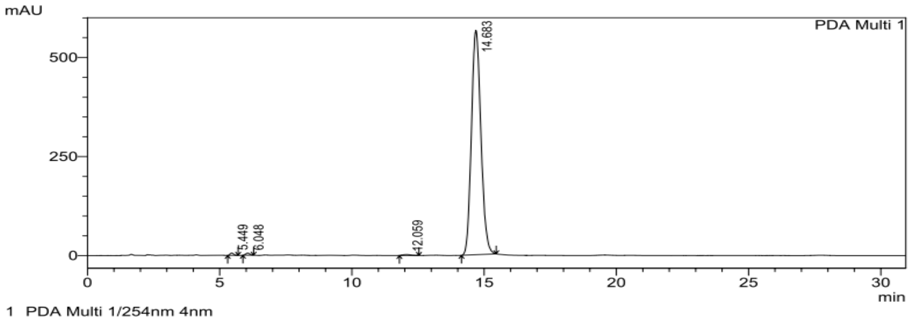

PeakTable

PDA Ch1 254nm 4nm

Peak#	Ret. Time	Area	Height	Area %	Height %
1	5.449	58406	6111	0.408	1.053
2	6.048	63630	5403	0.444	0.931
3	12.059	49454	2383	0.345	0.411
4	14.683	14143754	566399	98.802	97.605
Total		14315244	580296	100.000	100.000

Figure S24. HPLC Spectrum of compound **5a$_6$**.

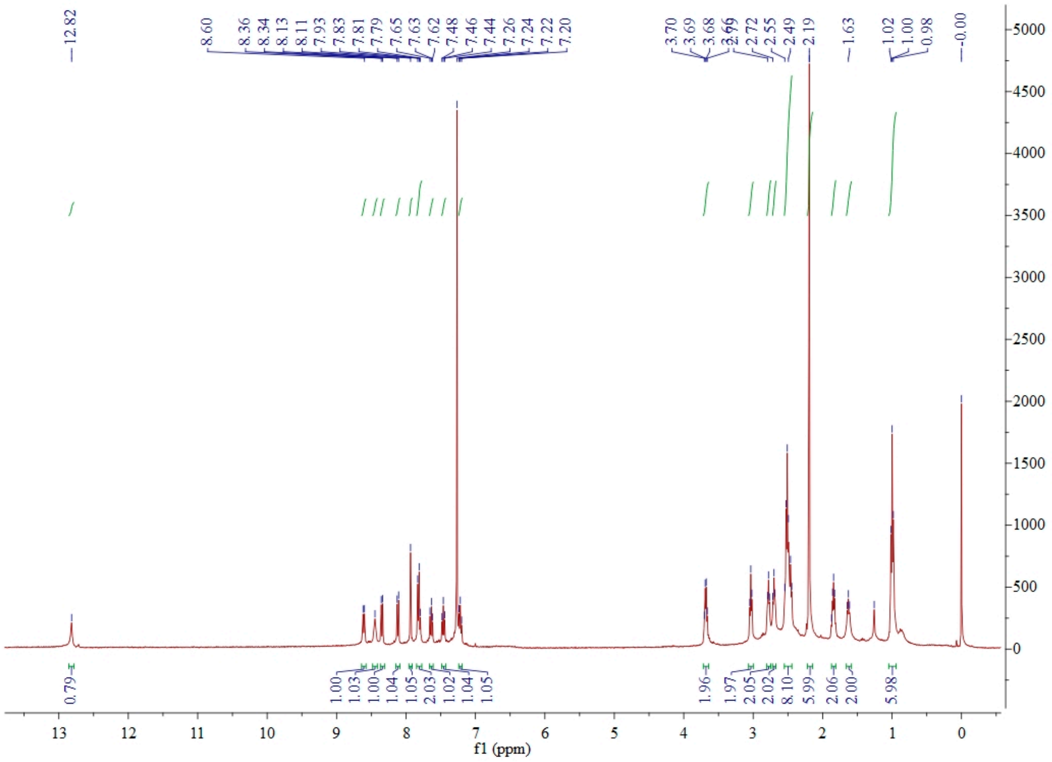

Figure S25. ^{1}H-NMR Spectrum of compound **5a**7.

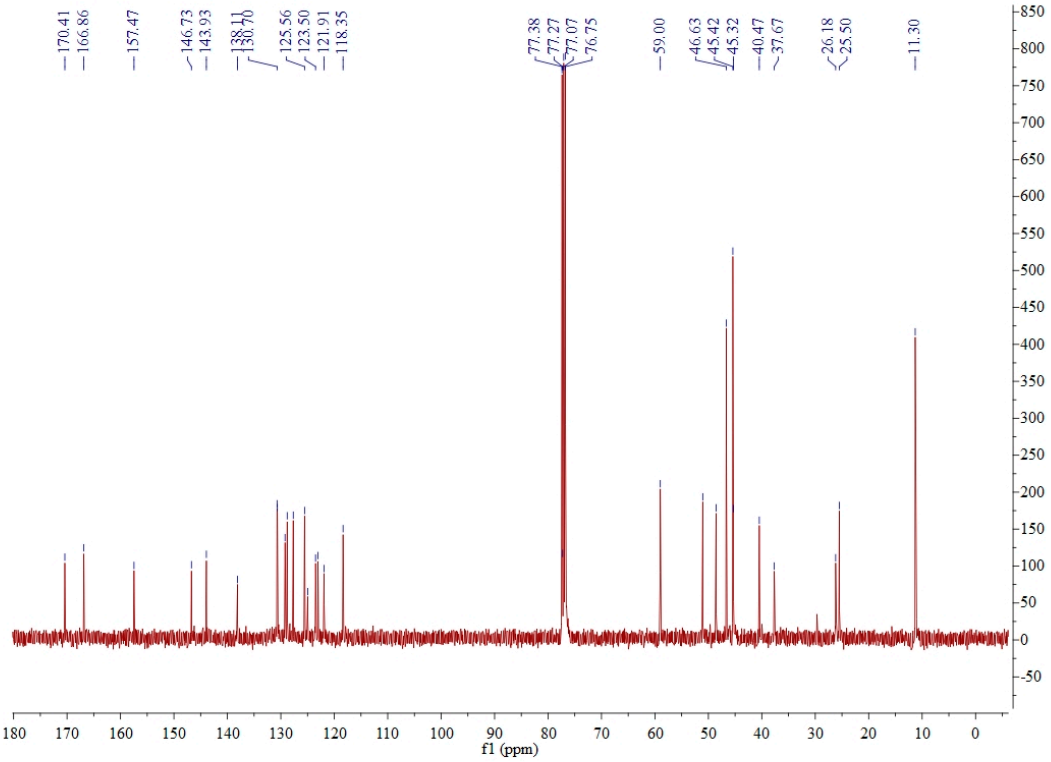

Figure S26. ^{13}C-NMR Spectrum of compound **5a**7.

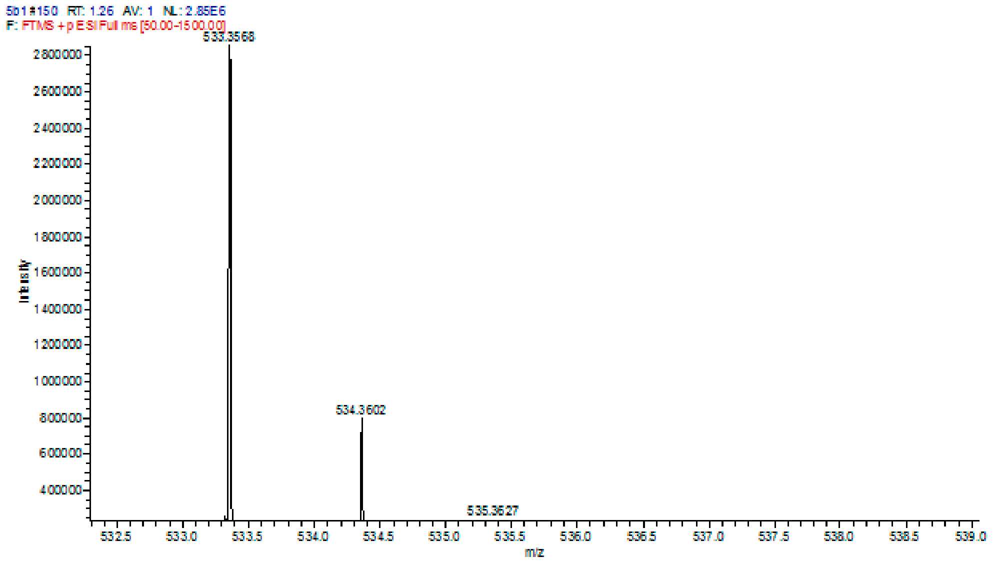

Idx	Formula	RDB	Delta mmu
1	$C_{31}H_{45}O_2N_6$	12.5	-3.041

Calculated 533.3599 Found 533.3568

Figure S27. HRMS Spectrum of compound **5a**7.

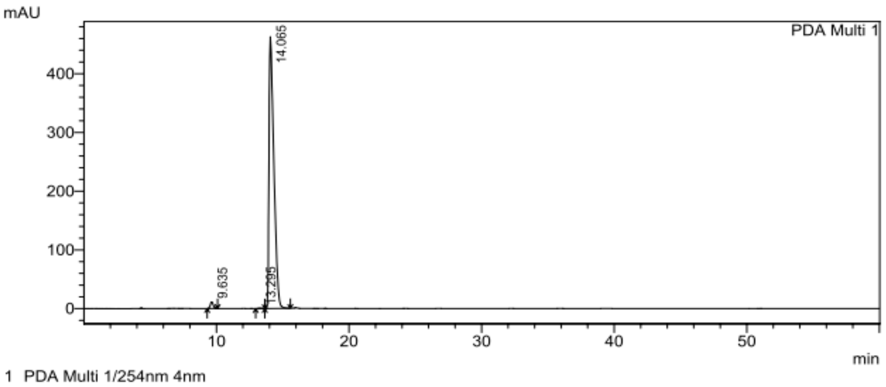

PeakTable

PDA Ch1 254nm 4nm

Peak#	Ret. Time	Area	Height	Area %	Height %
1	9.635	174187	11225	1.458	2.364
2	13.295	12076	660	0.101	0.139
3	14.065	11762079	462892	98.441	97.497
Total		11948341	474777	100.000	100.000

Figure S28. HPLC Spectrum of compound **5a**7.

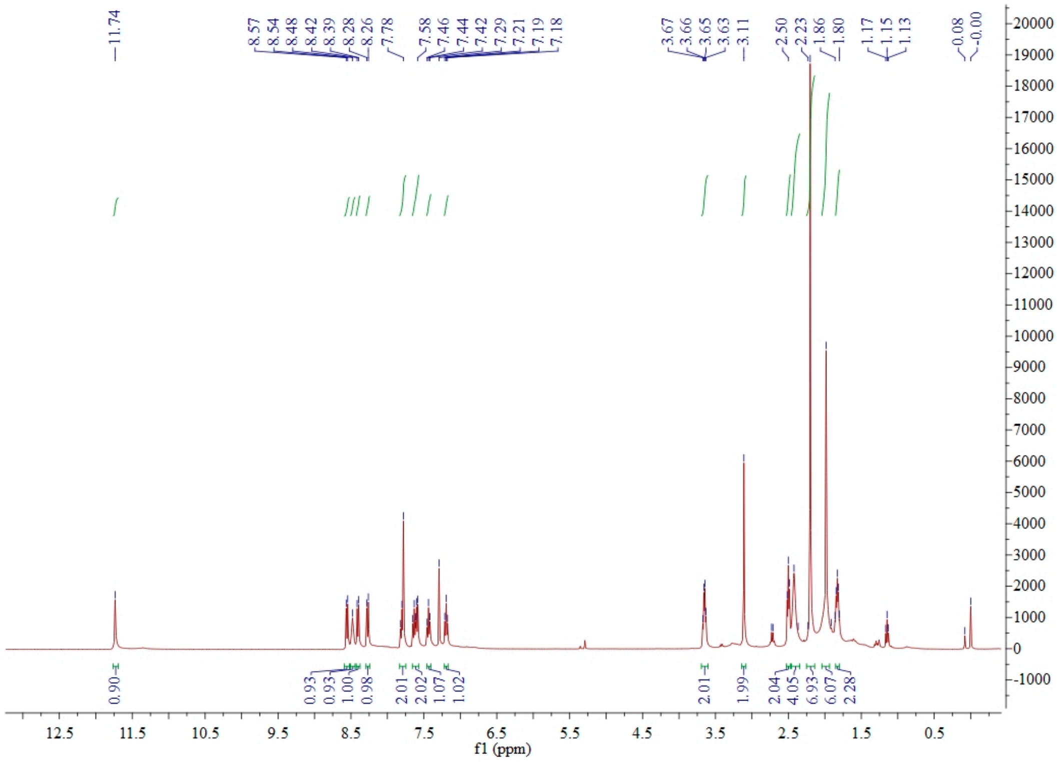

Figure S29. ^{1}H-NMR Spectrum of compound **5b**1.

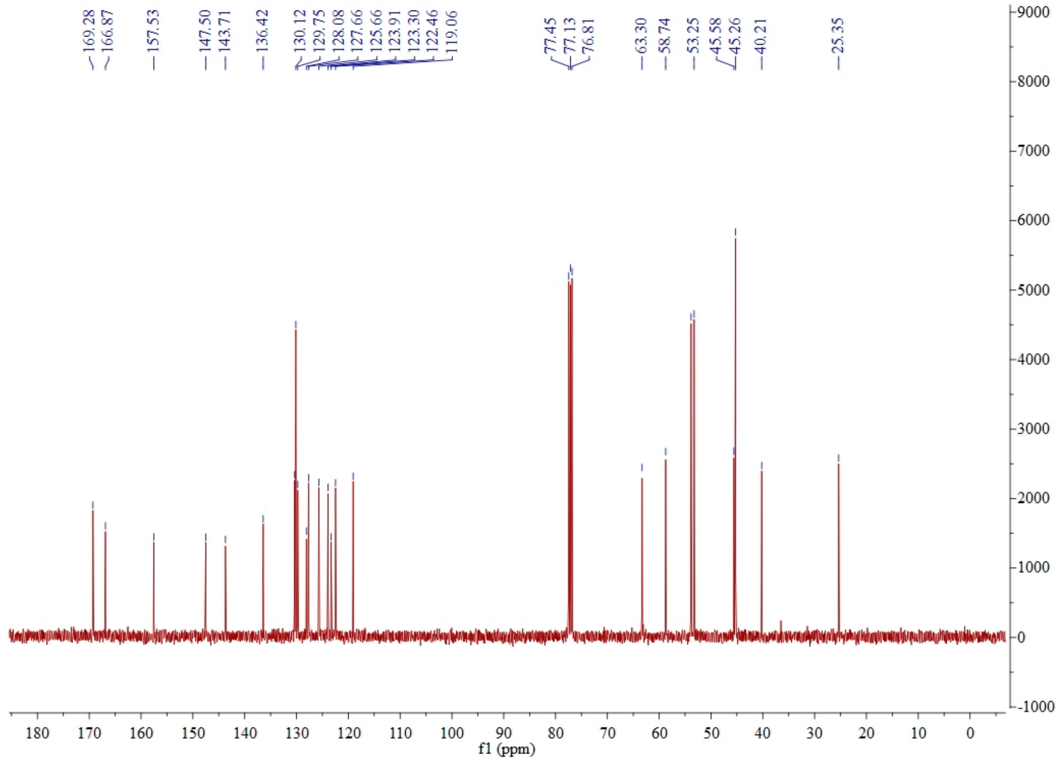

Figure S30. ^{13}C-NMR Spectrum of compound **5b**1.

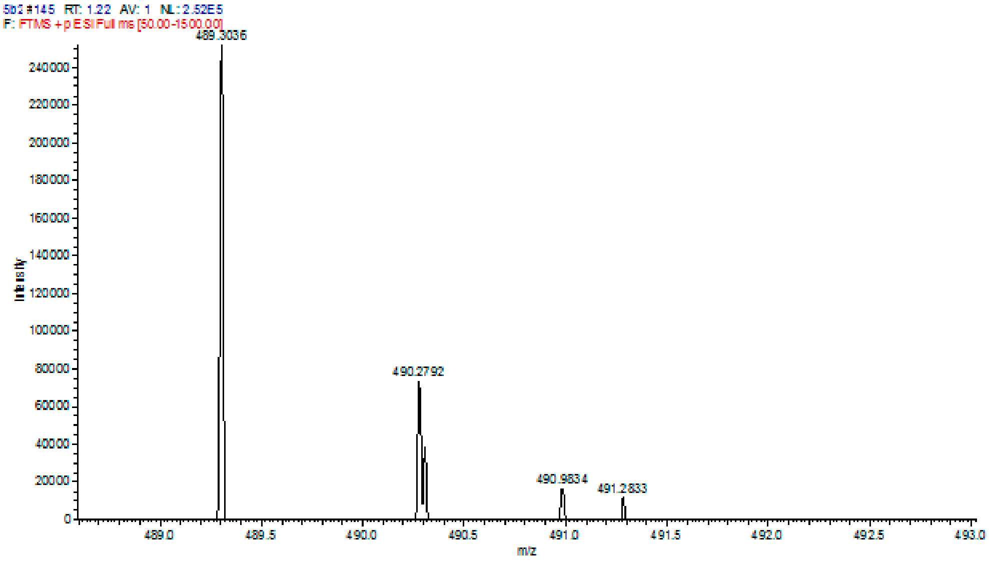

Idx	Formula	RDB	Delta mmu
1	$C_{28}H_{37}O_2N_6$	13.5	6.399

Calculated 489.2973 Found 489.3036

Figure S31. HRMS Spectrum of compound **5b**1.

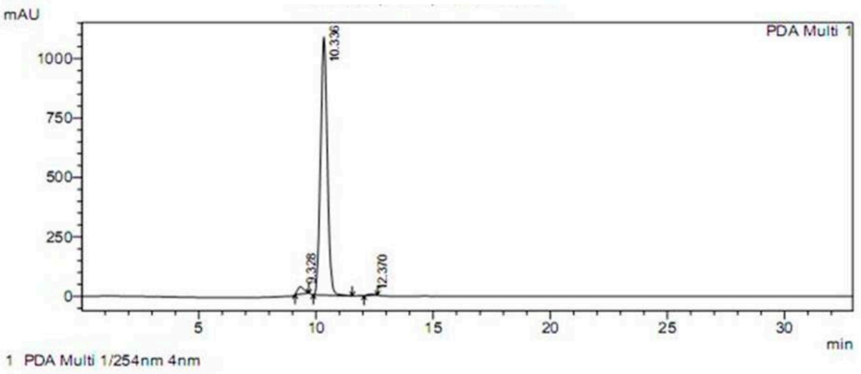

PeakTable

PDA Ch1 254nm 4nm

Peak#	Ret. Time	Area	Height	Area %	Height %
1	9.328	576203	30188	2.579	2.694
2	10.336	21664887	1085285	96.978	96.849
3	12.370	98901	5126	0.443	0.457
Total		22339991	1120599	100.000	100.000

Figure S32. HPLC Spectrum of compound **5b**1.

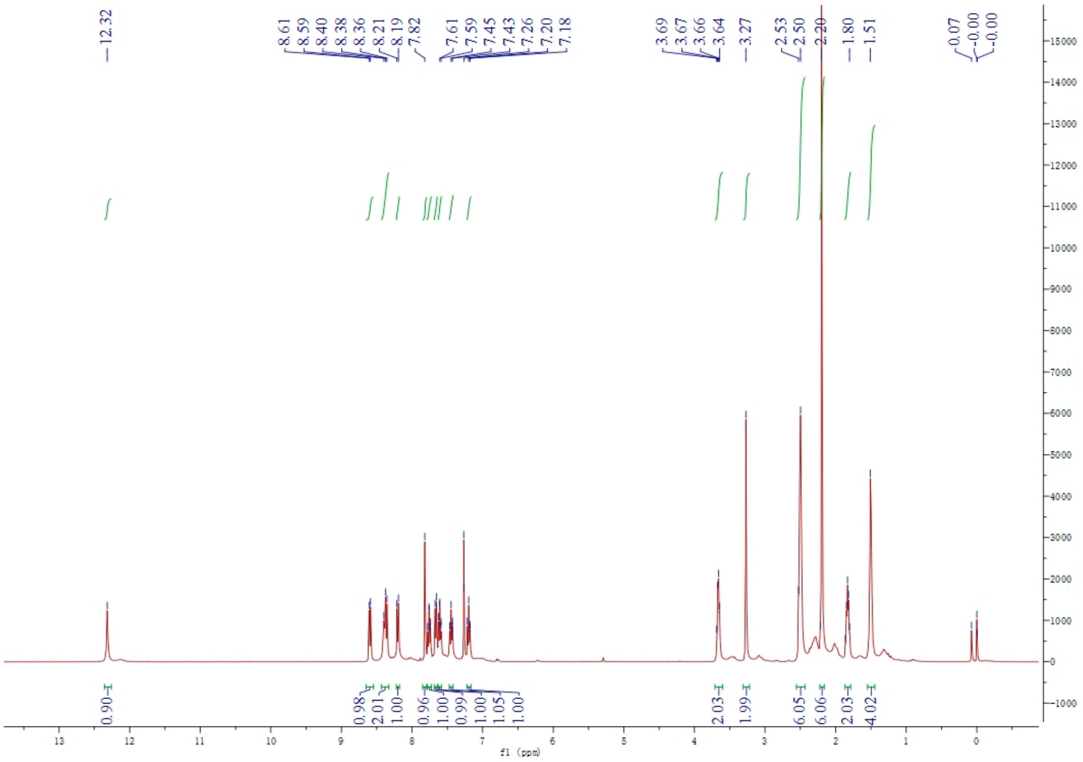

Figure S33. ^{1}H-NMR Spectrum of compound **5b**$_2$.

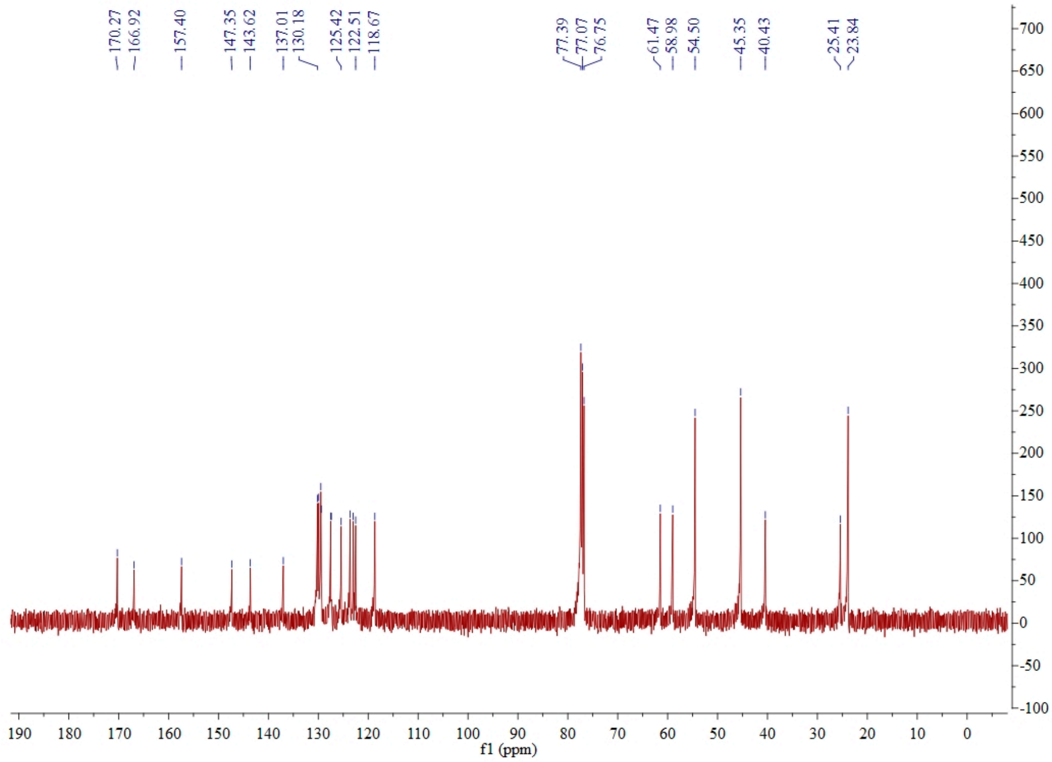

Figure S34. ^{13}C-NMR Spectrum of compound **5b**$_2$.

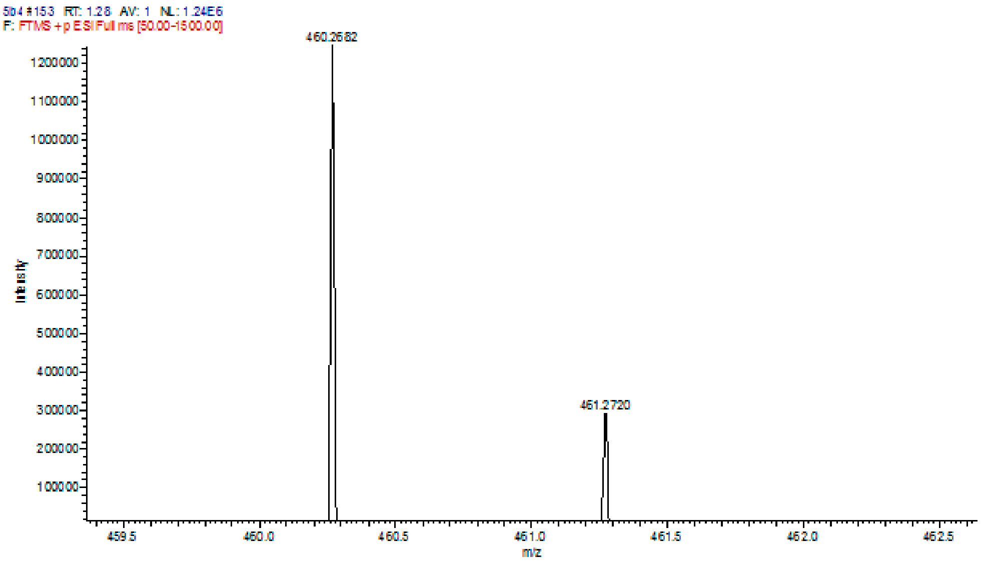

Idx	Formula	RDB	Delta mmu
1	$C_{27}H_{34}O_2N_5$	13.5	-2.542

Calculated 460.2707 Found 460.2682

Figure S35. HRMS Spectrum of compound **5b**2.

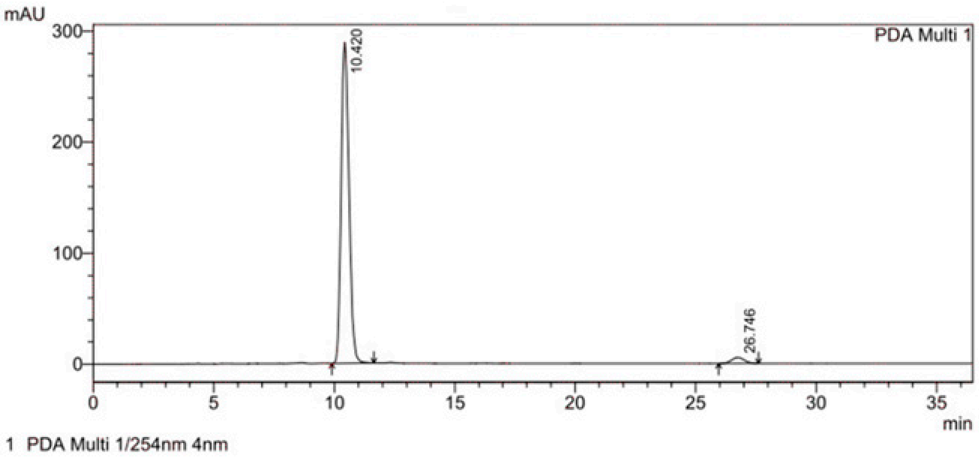

PeakTable

PDA Ch1 254nm 4nm

Peak#	Ret. Time	Area	Height	Area %	Height %
1	10.420	6566769	289073	97.022	98.189
2	26.746	201554	5333	2.978	1.811
Total		6768323	294406	100.000	100.000

Figure S36. HPLC Spectrum of compound **5b**2.

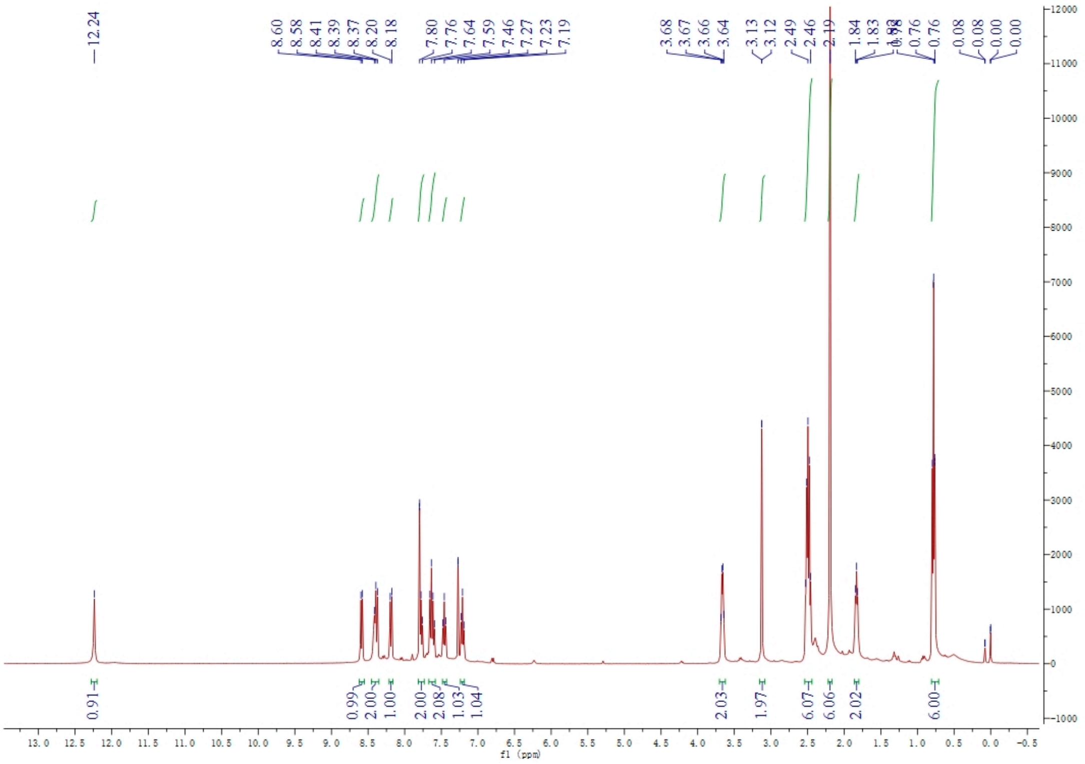

Figure S37. ^{1}H-NMR Spectrum of compound **5b**$_3$.

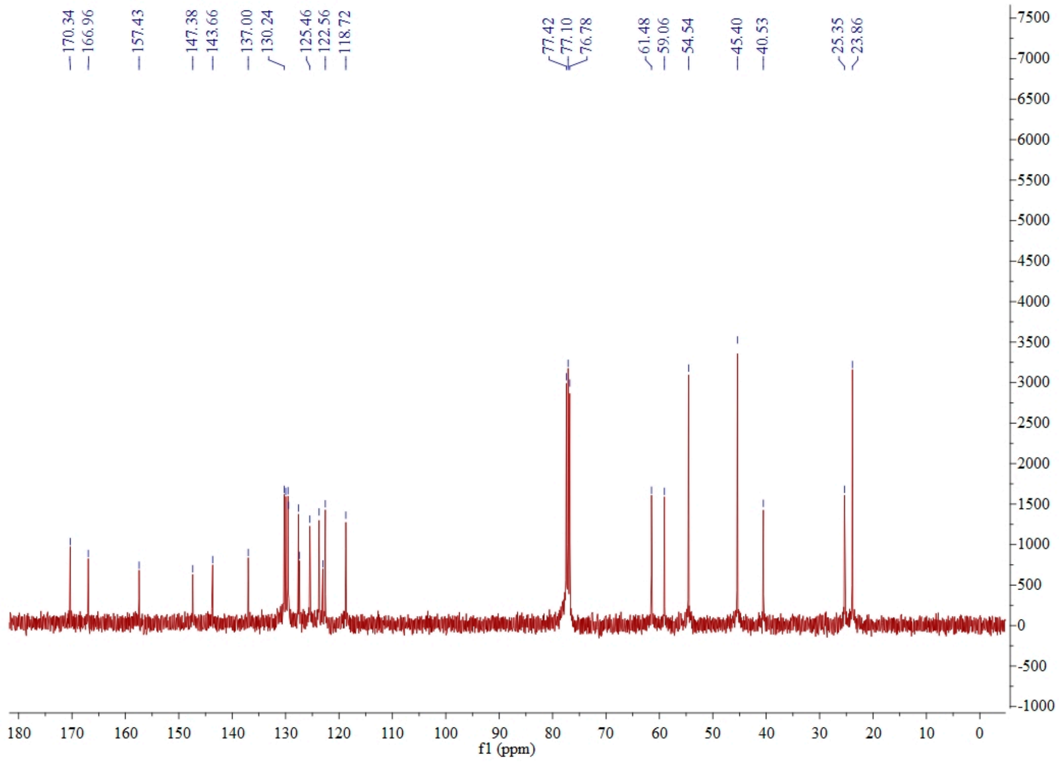

Figure S38. ^{13}C-NMR Spectrum of compound **5b**$_3$.

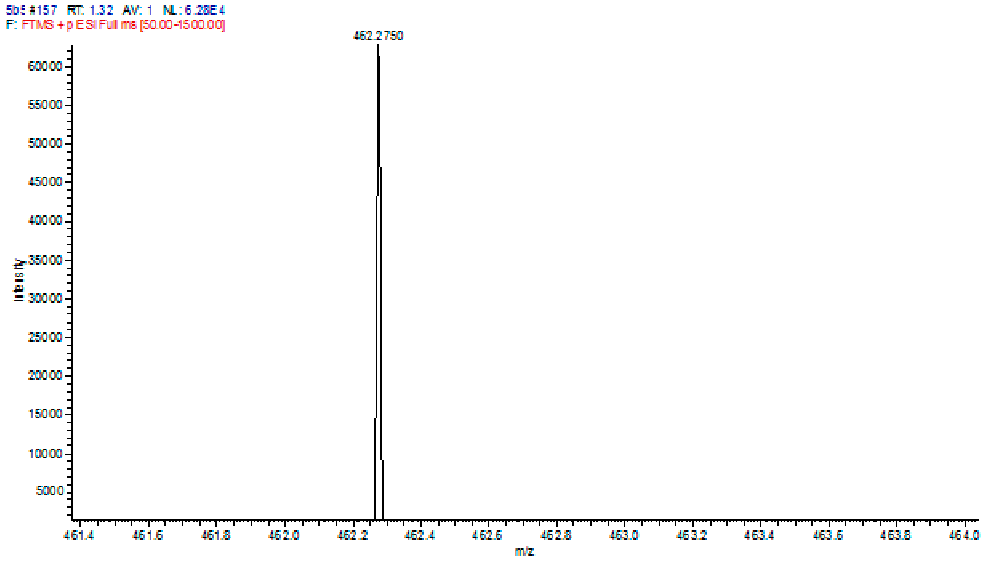

Idx	Formula	RDB	Delta mmu
1	$C_{27}H_{36}O_2N_5$	12.5	-11.332

Calculated 462.28647 Found 462.2750

Figure S39. HRMS Spectrum of compound **5b**3.

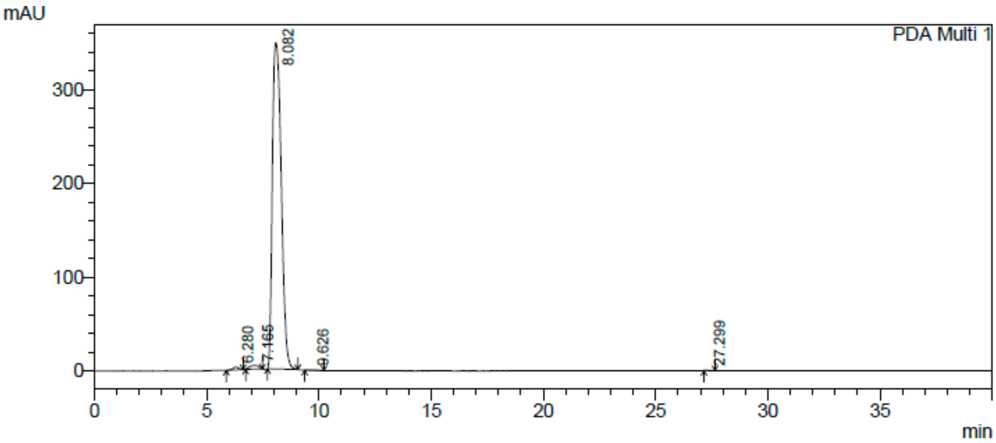

1 PDA Multi 1/254nm 4nm

PeakTable

PDA Ch1 254nm 4nm

Peak#	Ret. Time	Area	Height	Area %	Height %
1	6.280	56723	3155	0.581	0.887
2	7.165	110137	3808	1.129	1.071
3	8.082	9577029	348164	98.164	97.886
4	9.626	9209	372	0.094	0.105
5	27.299	3039	183	0.031	0.052
Total		9756136	355682	100.000	100.000

Figure S40. HPLC Spectrum of compound **5b**3.

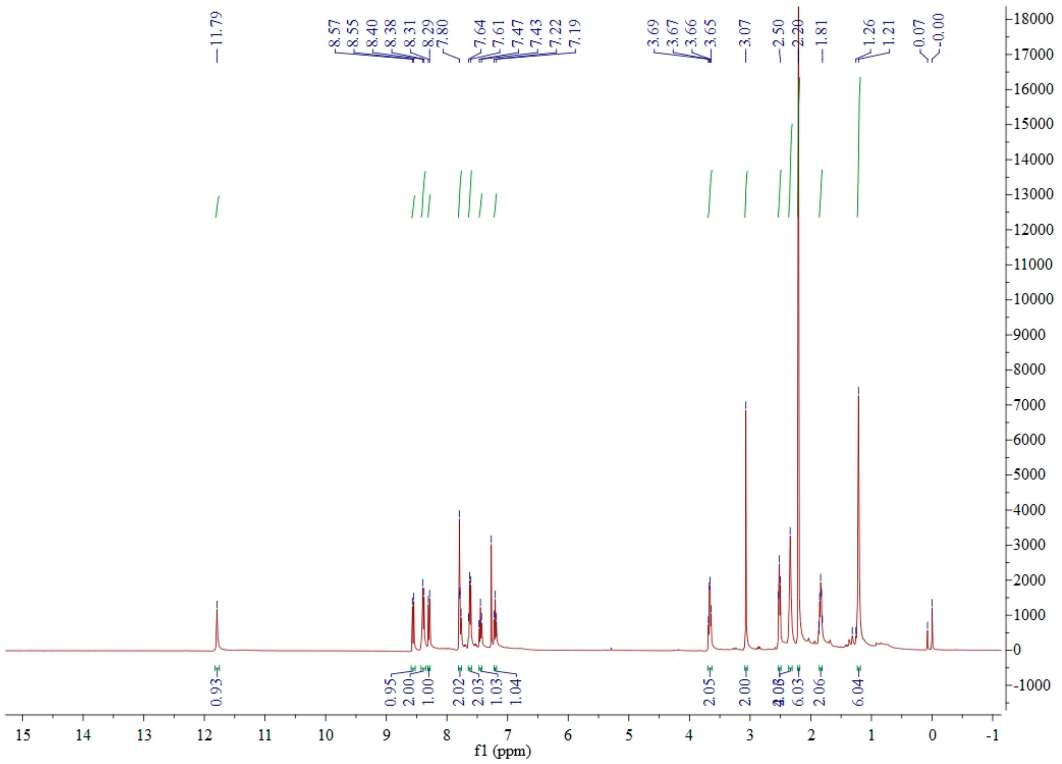

Figure S41. ^{1}H-NMR Spectrum of compound **5b**4.

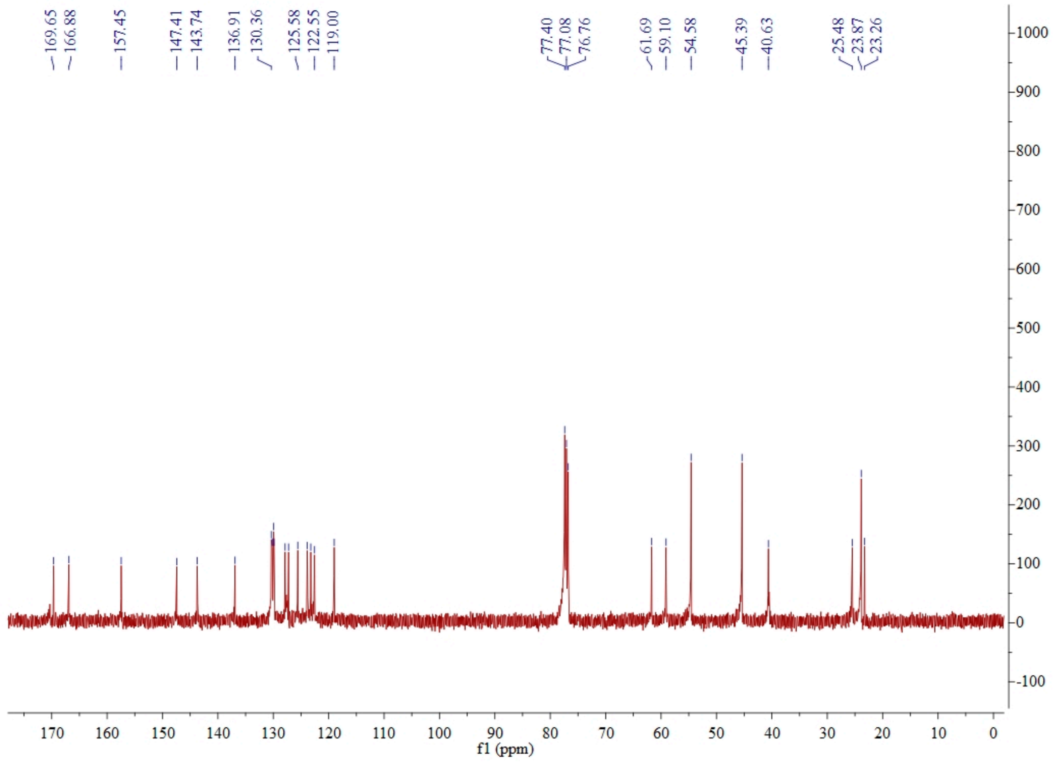

Figure S42. ^{13}C-NMR Spectrum of compound **5b**4.

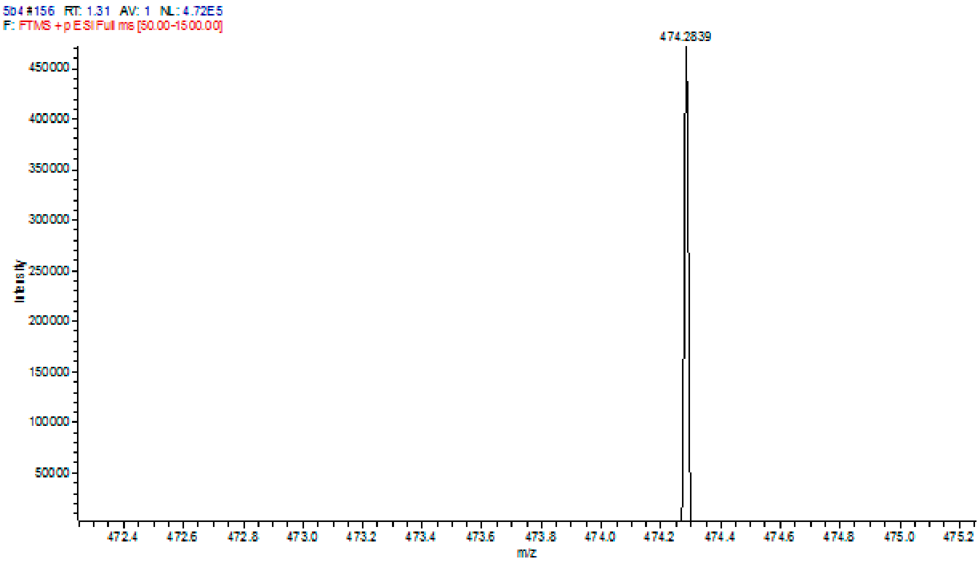

Idx	Formula	RDB	Delta mmu
1	$C_{28}H_{36}O_2N_5$	13.5	-2.482

Calculated 474.2864 Found 474.2839

Figure S43. HRMS Spectrum of compound **5b**4.

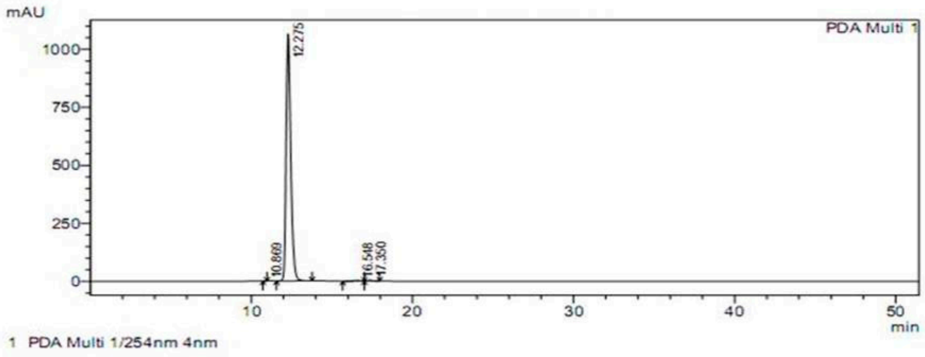

PeakTable

PDA Ch1 254nm 4nm

Peak#	Ret. Time	Area	Height	Area %	Height %
1	10.869	451	47	0.002	0.004
2	12.275	21237896	1065012	98.852	99.367
3	16.548	135893	2787	0.633	0.260
4	17.350	110326	3954	0.514	0.369
Total		21484567	1071799	100.000	100.000

Figure S44. HPLC Spectrum of compound **5b**4.

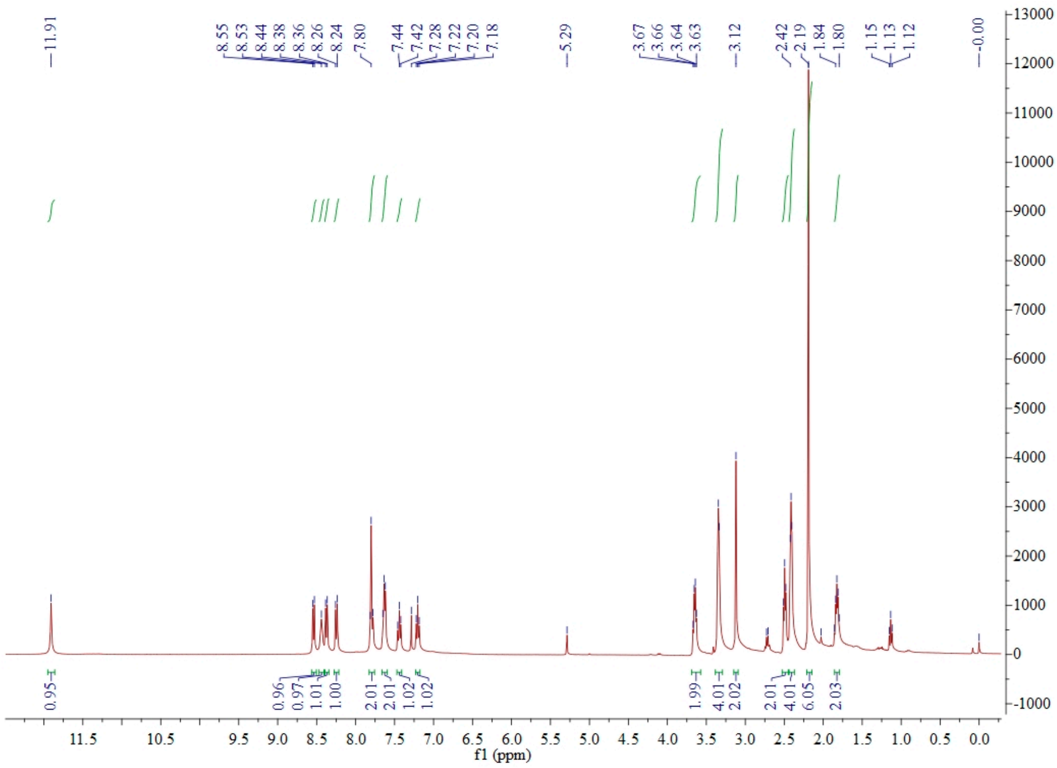

Figure S45. ^{1}H-NMR Spectrum of compound **5b**5.

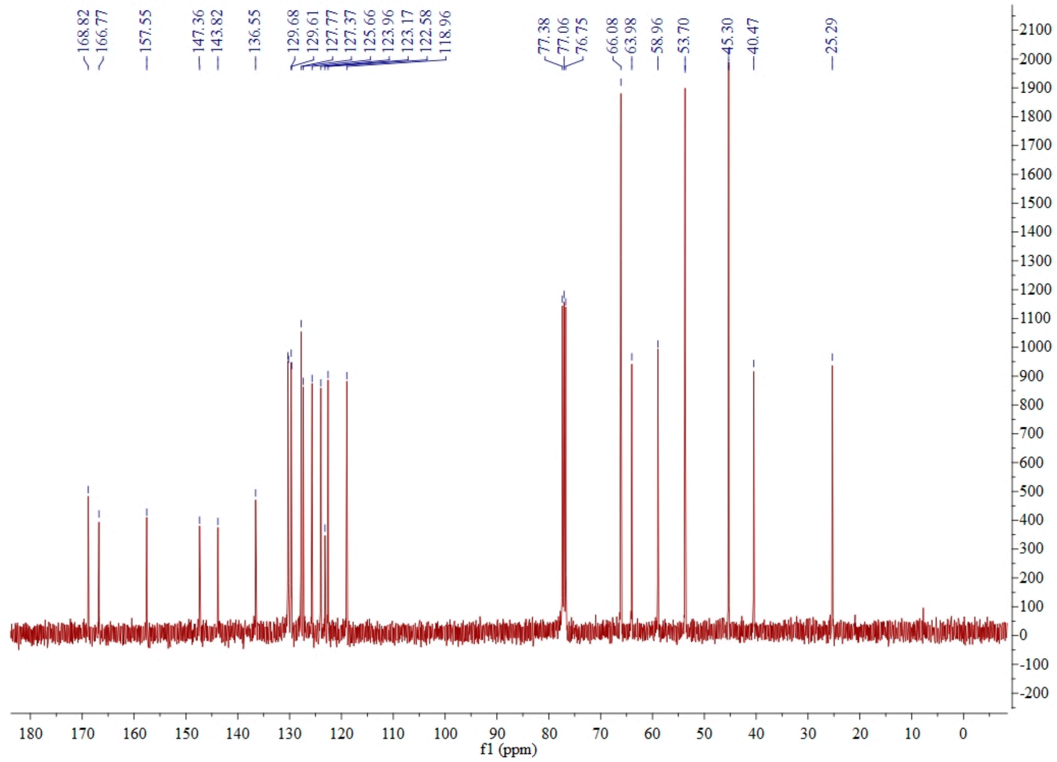

Figure S46. ^{13}C-NMR Spectrum of compound **5b**5.

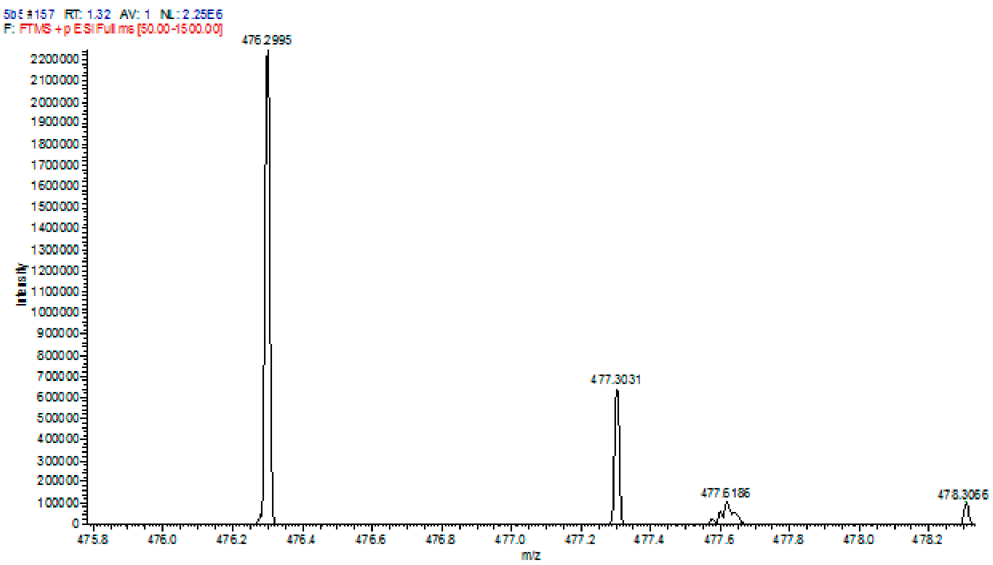

Idx	Formula	RDB	Delta mmu
1	$C_{27}H_{34}O_3N_5$	13.5	33.854

Calculated 476.2656 Found 476.2995

Figure S47. HRMS Spectrum of compound **5b**5.

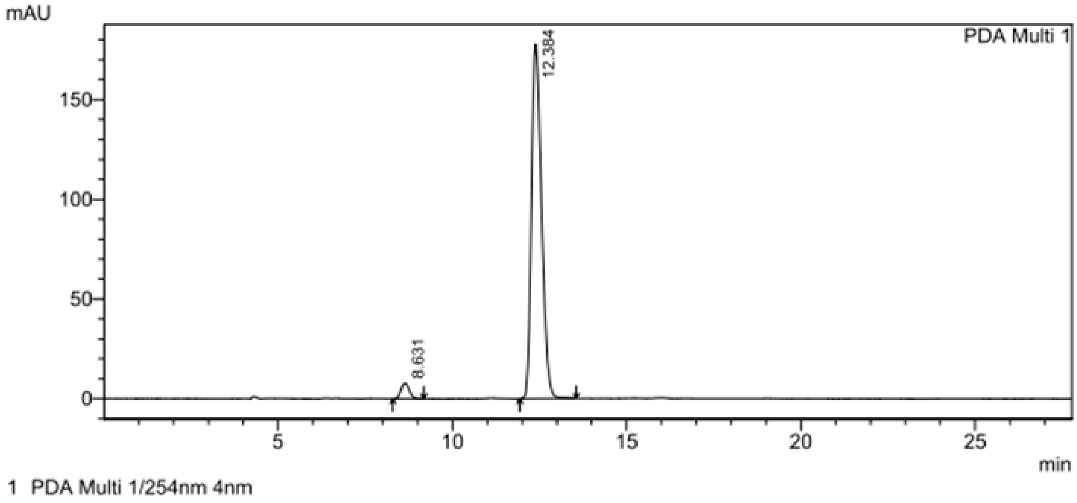

PeakTable

PDA Ch1 254nm 4nm

Peak#	Ret. Time	Area	Height	Area %	Height %
1	8.631	126388	7876	3.386	4.244
2	12.384	3606432	177683	96.614	95.756
Total		3732820	185559	100.000	100.000

Figure S48. HPLC Spectrum of compound **5b**5.

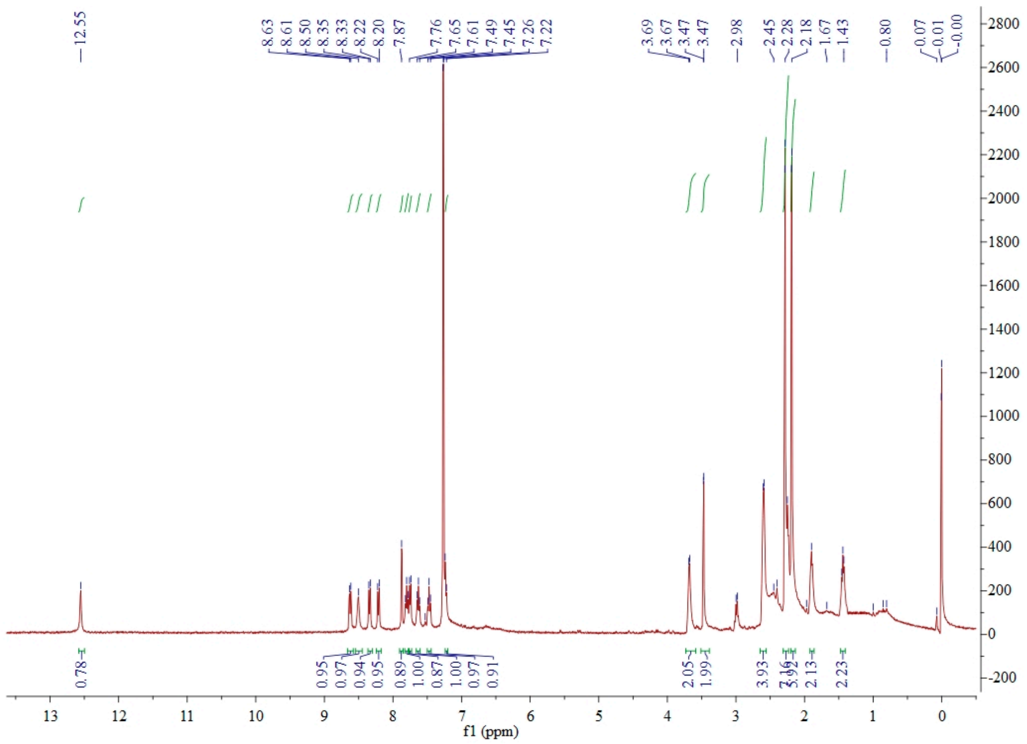

Figure S49. ^{1}H-NMR Spectrum of compound **5b**$_6$.

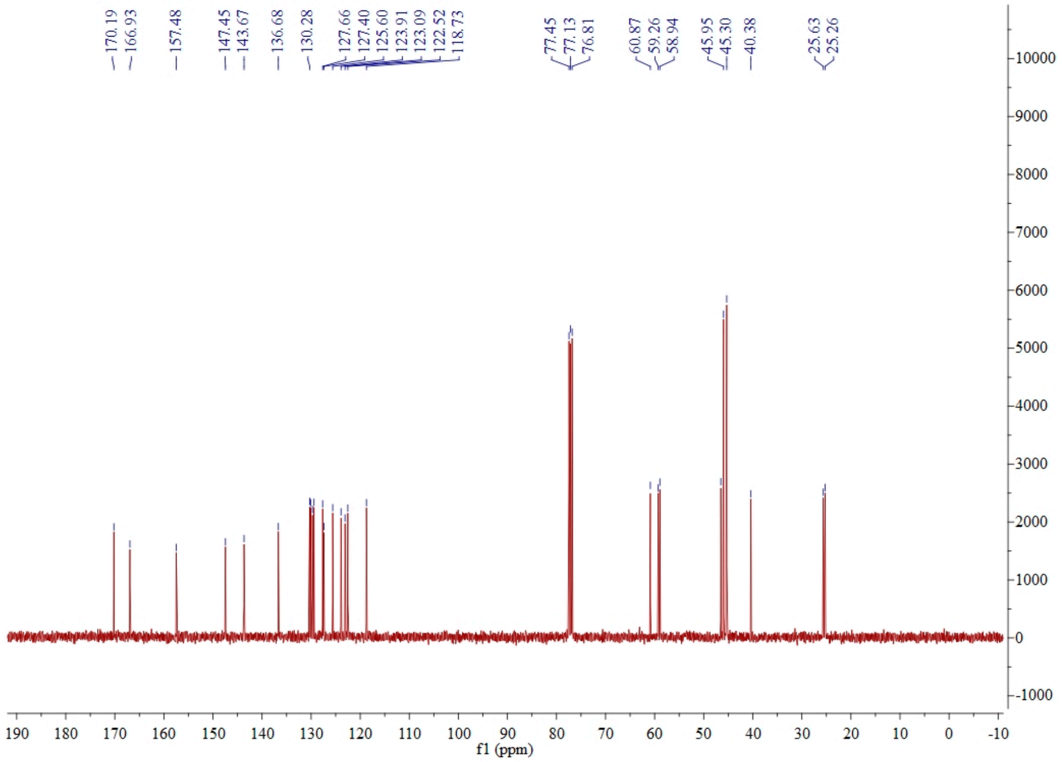

Figure S50. ^{13}C-NMR Spectrum of compound **5b**$_6$.

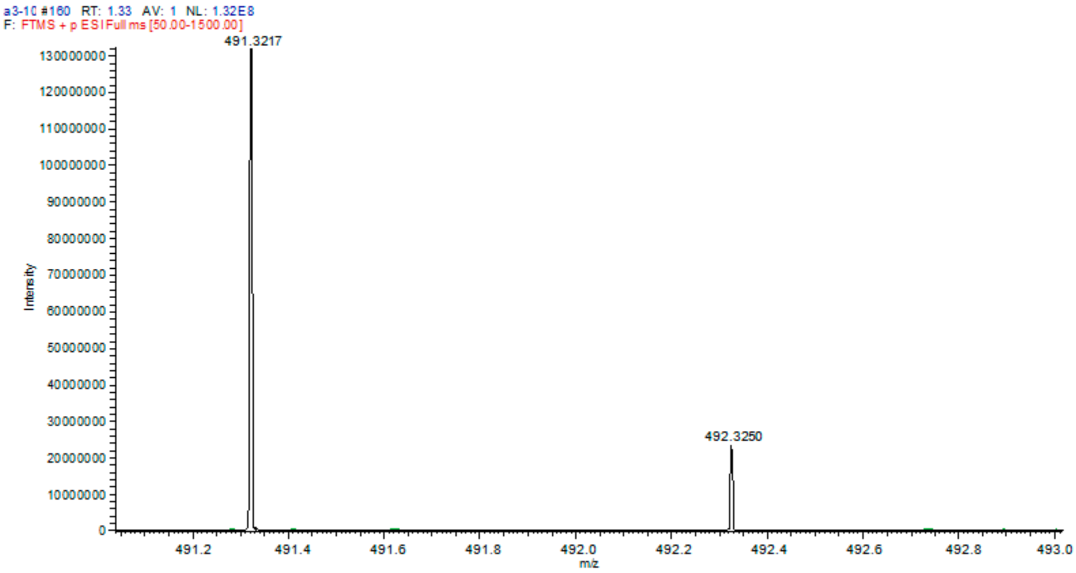

Idx	Formula	RDB	Delta mmu
1	$C_{28}H_{39}N_6O_2$	10.5	8.176

Calculated 491.3135 Found 491.3217

Figure S51. HRMS Spectrum of compound **5b**6.

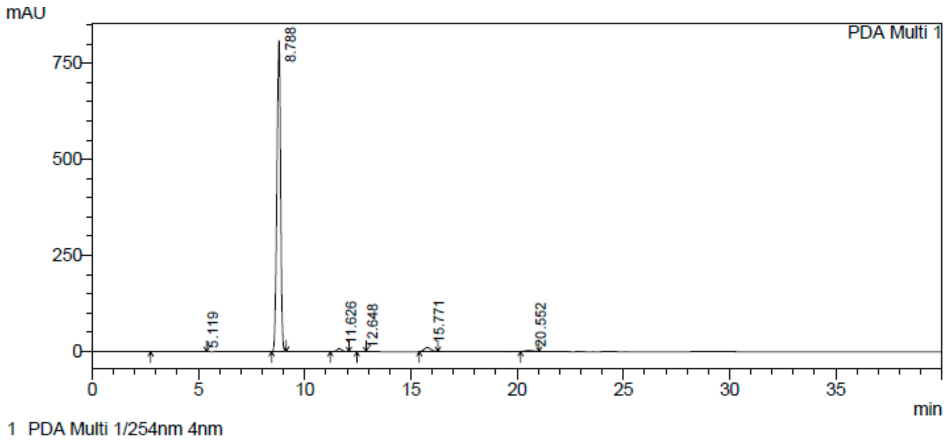

PeakTable

PDA Ch1 254nm 4nm

Peak#	Ret. Time	Area	Height	Area %	Height %
1	5.119	-11560	156	-0.114	0.019
2	8.788	9709566	809390	95.647	97.385
3	11.626	131880	7782	1.299	0.936
4	12.648	5474	427	0.054	0.051
5	15.771	232582	10334	2.291	1.243
6	20.552	83477	3037	0.822	0.365
Total		10151419	831127	100.000	100.000

Figure S52. HPLC Spectrum of compound **5b**6.
